# Nutritional factors and gender influence age-related DNA methylation in the human rectal mucosa

**DOI:** 10.1111/acel.12030

**Published:** 2012-12-06

**Authors:** Henri S Tapp, Daniel M Commane, D Michael Bradburn, Ramesh Arasaradnam, John C Mathers, Ian T Johnson, Nigel J Belshaw

**Affiliations:** 1 Institute of Food Research, Norwich Research ParkNorwich, NR4 7UA, UK; 2 Human Nutrition Research Centre, Institute for Ageing and Health, Campus for Ageing and Vitality, Newcastle UniversityBiomedical Research Building, Newcastle on Tyne, NE4 5PL, UK; 3 Wansbeck HospitalWoodhorn Lane, Ashington, UK

**Keywords:** aging, colorectal cancer, CpG islands, DNA methylation, folate, nutrition

## Abstract

Aberrant methylation of CpG islands (CGI) occurs in many genes expressed in colonic epithelial cells, and may contribute to the dysregulation of signalling pathways associated with carcinogenesis. This cross-sectional study assessed the relative importance of age, nutritional exposures and other environmental factors in the development of CGI methylation. Rectal biopsies were obtained from 185 individuals (84 male, 101 female) shown to be free of colorectal disease, and for whom measurements of age, body size, nutritional status and blood cell counts were available. We used quantitative DNA methylation analysis combined with multivariate modelling to investigate the relationships between nutritional, anthropometric and metabolic factors and the CGI methylation of 11 genes, together with LINE-1 as an index of global DNA methylation. Age was a consistent predictor of CGI methylation for 9/11 genes but significant positive associations with folate status and negative associations with vitamin D and selenium status were also identified for several genes. There was evidence for positive associations with blood monocyte levels and anthropometric factors for some genes. In general, CGI methylation was higher in males than in females and differential effects of age and other factors on methylation in males and females were identified. In conclusion, levels of age-related CGI methylation in the healthy human rectal mucosa are influenced by gender, the availability of folate, vitamin D and selenium, and perhaps by factors related to systemic inflammation.

## Introduction

Cytosine exhibits a nonuniform pattern of methylation in the human genome. Typically, Cytosine-Guanine dinucleotides (CpGs) distributed within both coding and noncoding regions are methylated, whereas those located within CpG islands (CGI), sequences with significant overrepresentation of CpGs and located within the promoter regions of more than 50% of mammalian genes, are usually unmethylated. There is compelling evidence for an association between colorectal carcinogenesis and aberrant DNA methylation (Lao & Grady, 2011), which includes both genome-wide loss of DNA methylation (global hypomethylation) and differential methylation of CGI of specific genes (CGI hypo- or hypermethylation). Global hypomethylation contributes to genome instability, whereas CGI hypo- and hypermethylation lead, respectively, to increased and decreased transcription of genes important for regulating processes disrupted during carcinogenesis (Jones & Baylin, 2002). Aberrant DNA methylation is observed in the early stages of neoplasia in the human colon (Chan *et al*., 2002) and also occurs in many apparently healthy tissues (Christensen *et al*., 2009), including the epithelial cells of the morphologically normal colonic crypt (Belshaw *et al*., 2010). Methylation of some CGI in the colorectal mucosa increases progressively with age, and this may contribute to field-wide changes in the colorectal mucosa that may increase its vulnerability to neoplastic transformation (Shen *et al*., 2005). In previous studies, we have quantified CGI methylation in biopsies from macroscopically normal colorectal mucosa, and confirmed that field methylation patterns differ significantly between healthy subjects and patients with neoplastic lesions (Belshaw *et al*., 2008). We have also shown that methylation levels measured within mucosal biopsies reflect highly variable levels of methylation in individual crypts (Belshaw *et al*., 2010). This mosaic pattern of CGI methylation in the mucosal field is probably a consequence of the unique methylation signatures of the corresponding stem cells.

The origins of aberrant CGI methylation are uncertain but, if the phenomenon plays a functional role in colorectal carcinogenesis, associations between CGI methylation and environmental factors known to influence cancer risk would be anticipated. The most important risk factor for colorectal carcinogenesis is advancing age (Armitage & Doll, 1954), and significant associations between age- and gene-specific CGI methylation have been reported (Belshaw *et al*., 2008). Apart from age, CGI methylation may be influenced by diet, metabolic status or other environmental factors (Christensen *et al*., 2009; Talens *et al*., 2012). In a recent study, Wallace *et al*. measured the methylation status of CGI in *ESR1* and *SFRP1* in biopsies from the right colon and rectum of 389 polyp patients enrolled in a multicentre trial of aspirin and folic acid for prevention of polyp recurrence (Wallace *et al*., 2010). For both genes, methylation varied with ethnicity, and with anatomical location, and positive associations were observed with age and red cell folate (RCF) status.

In the cross-sectional study described here we have quantified the CGI methylation status of 11 genes, together with LINE-1 as an index of global DNA methylation, in rectal biopsies from 185 patients undergoing diagnostic colonoscopy, who were shown to be free of gastrointestinal inflammatory or neoplastic disease. The panel of genes was selected on the basis of previous studies showing age- and cancer-associated methylation, or a cancer-related function or that methylation status contributed to the discrimination of normal mucosa from patients with and without neoplasia (Belshaw *et al*., 2008; Lao & Grady, 2011). Multivariate modelling using a genetic algorithm approach was used to assess relationships between DNA methylation and age, anthropometry, nutrient status and blood cell counts. We confirmed a strong positive effect of age on CGI methylation levels in the normal rectal mucosa. In addition, we identified effects of gender, and showed a significant positive association between CGI methylation levels and folate status and negative associations with selenium and vitamin D. We also obtained evidence for significant effects of white blood cell (WBC) counts and anthropometric factors.

## Results

**Figure 1:**
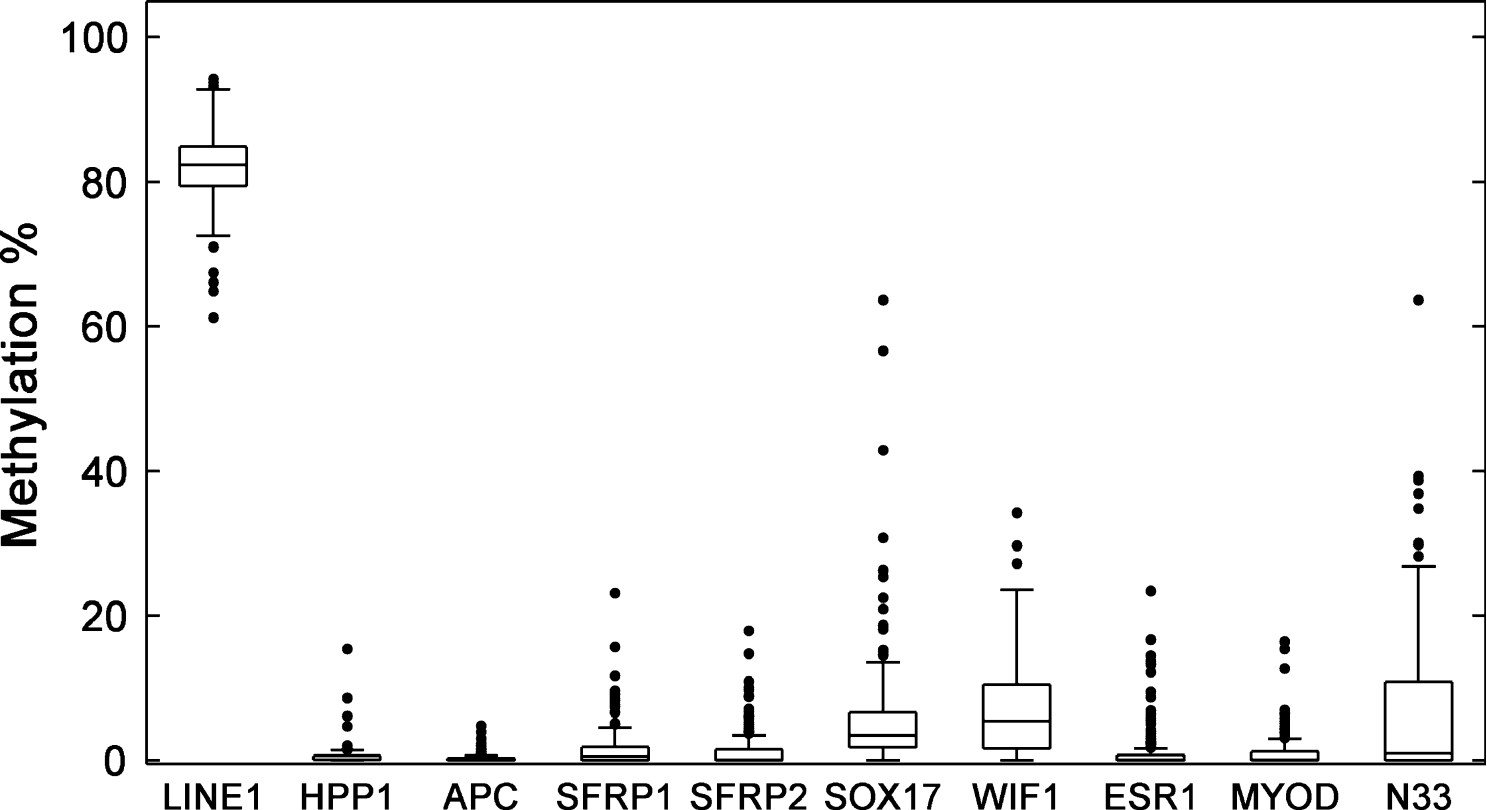
Summary descriptions of methylation values for the nine genes and for LINE-1.

The participant characteristics are summarized in Table 1 and correlations between each cofactor are provided in Supplementary Table S1. Methylation characteristics of the nine gene-specific CGI and of LINE-1 (Fig 1) show clear differences in the mean methylation and in the extent of interperson variability between different CGIs. Methylation of *AXIN2* and *DKK1* was generally very low and showed no correlation with age (data not shown) so these genes were excluded from subsequent analysis. For the remaining genes, seven showed strong positive intercorrelations (unadjusted *P* < 0.001), indicating concomitant methylation of these genes within a given participant (Supplementary Table S2). An eighth gene, *N33*, was correlated only with *SOX17* and *WIF1* (*P* < 0.01) while none of the correlations with *APC* were significant at the *P* = 0.001 level. LINE-1 methylation was uncorrelated with all nine genes (unadjusted *P* > 0.05).

**Table 1:** Characteristics of the study participants

Factor	All	*n* = 185		Men	*n* = 84		Women	*n* = 101	
Min	Median	Max	Min	Median	Max	Min	Median	Max
Age (years)	17	50	77	17	45	77	18	52	76
Height (m)	1.49	1.68	1.98	1.62	1.79	1.98	1.49	1.62	1.75
Weight (kg)	45	80	163	50	88	163	45	72	125
BMI (kg/m^2^)	17	28	51	17	28	50	19	27	51
Waist (cm)	64	93	150	65	99	150	64	86	118
Hip (cm)	80	103	143	84	103	143	80	102	141
Waist:Hip ratio (WHR)	0.71	0.88	1.13	0.77	0.95	1.13	0.71	0.83	0.99
Red cell folate (RCF) (ng/mL)	77	336	922	77	382	815	154	321	922
Plasma folate (PF) (nmol/L)	1.00	6.30	18.10	1.00	6.30	14.60	2.70	6.50	18.10
Homocysteine (μmol/L)	3.22	9.52	63.90	3.22	11.70	63.90	4.69	8.77	47.48
White cell count (WC) (×10^6^/mL)	3.50	7.60	16.70	3.50	7.50	15.70	4.10	7.60	16.70
Monocyte count (MC) (×10^3^/mL)	0.12	0.52	1.30	0.21	0.54	1.30	0.12	0.49	1.23
Vitamin D (vitD) (nmol/L)	19	73	258	23	68	224	19	75	258
Selenium (Se) (μmol/L)	0.61	1.07	1.87	0.64	1.07	1.73	0.61	1.09	1.87
Fatness Index (FI) (%)	5.8	31.6	49.7	5.8	27.0	38.3	22.1	36.6	49.7
Vitamin B12 (vitB12) (pmol/L)	110	372	1133	125	359	953	110	377	1133

The GA-ANCOVA (genetic algorithm with analysis of covariance) modelling aimed to find subsets of exposure variables that best explained the variation in methylation, allowing particular attention to be given to variables that occurred frequently within the subset models. The final models, the frequency of occurrence over 10 GA evaluations, and the square of the correlation coefficient between actual and noncross-validated predictions, which represents the fraction of explained variance, are given in Table 2. The genes for which the measured exposure factors explained the highest and lowest proportion of the variance were *WIF1* (> 30%) and *APC* (< 5%), with the remaining genes and LINE-1 having values in the range 10–20%. The model for the summary statistic PCA1 (the first principal component score vector) explained the largest proportion of the observed variance (38%).

**Table 2:** Models which predicted methylation of the nine genes, LINE-1 and PCA1

Gene	*n*	ANCOVA Terms	n1CV	*R*^2^	2CV% Explained Variance (95% CI)
*WIF1*	185	Age, MC, Se, WC, BMI, PF	7	0.302	17.1 (8.2–27.7)
*HPP1*	185	Age, sex^*^MC, sex^*^age, sex, MC	9	0.189	5.0 (0.7–12.7)
*APC*	185	vitD, FI, age	7	0.048	–
*SFRP1*	185	Age, sex^*^RCF, sex, RCF, MC	10	0.197	4.3 (0.4–11.6)
*SFRP2*	185	Age, FI	9	0.104	3.9 (0.3–11.0)
*SOX17*	185	age, sex^*^age, sex, WC	9	0.167	5.6 (0.9–13.6)
LINE-1	185	Hip, sex^*^height, Se, BMI, vitB12, vitD, height, sex	8^a^	0.142	–
*ESR1*	174	Age, sex^*^height, height, sex	9	0.181	8.0 (1.9–17.1)
*MYOD*	174	Age, PF, vitD	8	0.170	7.1 (1.5–15.9)
*N33*	174	Age, sex^*^PF, sex^*^waist, sex, waist, PF	3	0.131	–
PCA1	174	Age, PF, sex^*^age, sex, Se, vitD	9^a^	0.380	21.2 (11.2–32.5)

*n*, number of subjects in data set.

ANCOVA, analysis of covariance, an ^*^ between cofactors indicates a sex interaction.

Terms ordered by their significance in the model: BMI, body mass index; FI, fatness index; MC, monocytes; PF, plasma folate; RCF, red cell folate; Se, selenium; vitB12, vitamin B12; vitD, vitamin D; WC, white cells.

n1CV, number of occurrences of the model in 10 epochs, ^a^ denotes 1 tie.

*R*^2^, square correlation between actual and predicted values (no cross-validation).

2CV% Explained Variance: estimate based on double cross-validation of variance explained if model was applied to new cohort. Missing entries signify the model failed double cross-validation.

The selection criteria may be susceptible to some degree of overfitting and hence the *R*^2^ figures in Table 2 are optimistic estimates of explained variance. The ability to generalize was therefore evaluated using double cross-validation (2CV). This appraised the overall modelling process as well as the robustness of modelling data associated with a particular gene. The models for predicting methylation of *WIF1, HPP1, SFRP1, SFRP2, SOX17, ESR1, MYOD1,* and PCA1 all passed 2CV, suggesting that they could generalize to other datasets. The models for *APC, N33,* and LINE-1 methylation failed 2CV, probably because of overfitting, and caution should be exercised in their interpretation. As before, the PCA1 model captured the most methylation variance (21.1%), providing further evidence for concordance in CGI methylation in response to age and other exposures.

**Figure 2:**
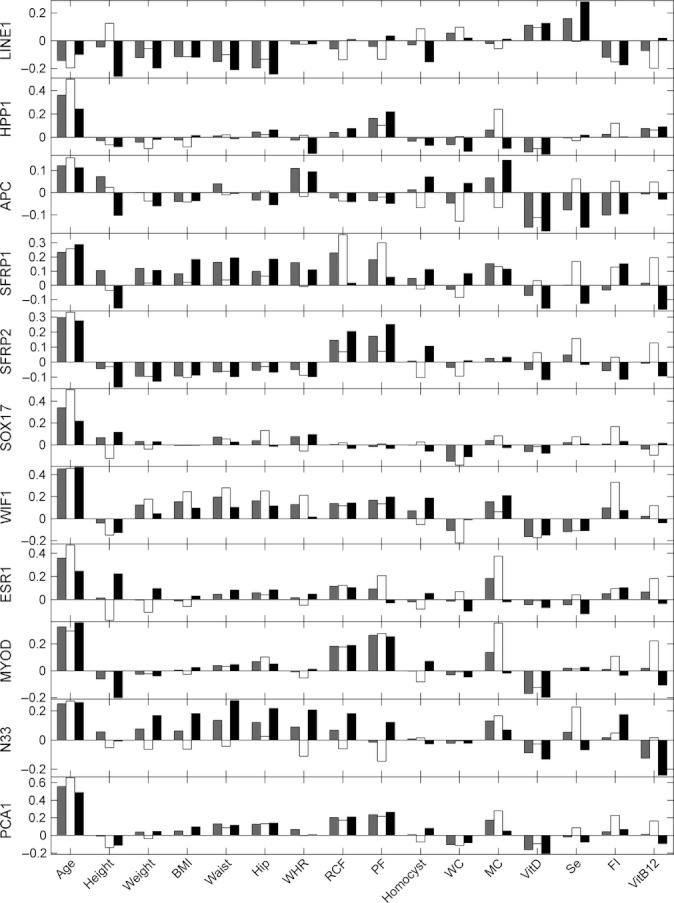
Pearson correlation coefficients for the associations between the exposure variables and the methylation of the nine genes, LINE-1 and PCA1 for all subjects (grey), males only (white) and females only (black).

For each of the models (Table 2), the corresponding ANCOVA tables, regression vectors and a graphical representation of the models are provided as supplementary material (Supplementary GA-ANCOVA file). Figure 2 summarizes the Pearson correlation coefficients between each of the exposure variables and the methylation of each gene, LINE-1 and PCA1. The correlations are shown separately for all study participants, males only, and females only (further details in Supplementary Tables S3a–c).

## Age

There was a strong positive correlation between participant age and PCA1 methylation (*r* = 0.556), and the GA model also identified an interaction between age and gender. Inspection of the age regression coefficients showed that these were positive for both genders but larger in males. Figure 2 shows that age was also correlated positively in both genders but stronger in males. This correspondence was found in *HPP1* and *SOX17*, also modelled with sex interactions (Table 2). The difference in the age regression coefficients may therefore reflect a lower weighting of age in females due to their poorer correlation. The link between methylation and only age and gender was investigated using ANCOVA for PCA1, *HPP1* and *SOX17*. In all cases, the sex interaction term was significant, the gradients were positive for both genders and significantly larger in males.

**Figure 3:**
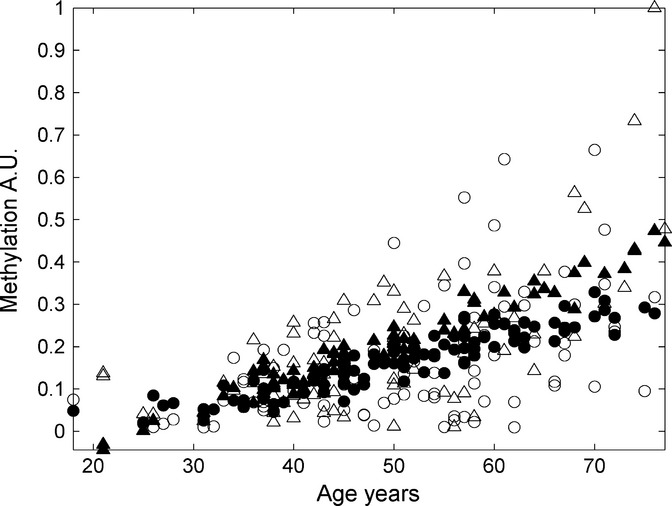
PCA1 methylation in the male (Δ) and female (○) subjects calculated from the CGI methylation values for the nine genes (open symbols) and predicted from the GA model (filled symbols).

Figure 3 shows the variation in methylation with age for PCA1 but the same general behaviour was seen for all of the genes studied (Supplementary GA-ANCOVA file). Figure 3 shows a characteristic ‘wedge-shape’, or *heteroscedastic*, response with age, in which the mean and range of methylation increased with age. This observation violates the assumption of homoscedastic variance made in the regression analysis. The correspondence between the sizes of the correlation and gradient coefficients for gender-related age effects on methylation may therefore be coincidental but, alternatively, could be due to the heteroscedastic relationship between age and methylation. Considering the gradient and correlation findings separately, the steeper gradient in males implies that older males have higher methylation than females of the same age. A lower correlation with age in females suggests that other factors have a greater influence on their CGI methylation. Figure 2 shows that the stronger correlation of age with methylation in males was also seen to lesser extents in *ESR1, APC* and *SFRP2*. For LINE-,1 there was a weak negative correlation between age and methylation, consistent with the global loss of DNA methylation during aging (Iacopetta *et al*., 2007).

## Nutritional status

Plasma folate (PF) status was selected in the PCA1 methylation model (Table 2), and was correlated positively with methylation (Fig 2). Folate status, either PF or RCF, was also selected in the models for *WIF1, MYOD1, SFRP1* and *N33* methylation (Table 2), and for *SFRP1* and *N33* there was an interaction between folate status and gender. *SFRP1* methylation correlated positively with RCF only in males whereas *N33* was positively correlated with PF in females and negatively in males. LINE-1 methylation showed a weak negative correlation with folate status in males.

The selection of plasma selenium in the PCA1 methylation model (Table 2) reflected a consistent trend, observed for several of the genes, of a positive correlation with CGI methylation in males and a negative correlation in females (Fig 2). However, for *WIF1* methylation*,* the correlation with plasma selenium was negative in both males and females (Fig 2). There was a positive correlation between LINE-1 methylation and plasma selenium only in females (*r* = 0.283, unadjusted *P* < 0.01).

Plasma vitamin D was associated with PCA1 methylation (Table 2). In general, vitamin D status and gene-specific methylation were correlated negatively, with a trend towards stronger associations in females, whereas a small positive correlation with LINE-1 methylation was observed for both genders (Fig 2).

Neither vitamin B-12 nor homocysteine was selected by any of the models of gene-specific methylation, although there was a weak correlation with *MYOD1* methylation in males (*r* = 0.223, unadjusted *P* < 0.05). Vitamin B-12 was selected in the model for LINE-1 methylation (Table 2) with a nonsignificant negative correlation in males (*r* = −0.199) and no association in females (*r* = 0.017) (Fig 2). Homocysteine was not significantly correlated with methylation of any of the genes, or with LINE-1.

## Anthropometry

Measures of body size including body mass index, FI (a derived measure of body adiposity), waist and hip circumferences and height did not feature in the model for PCA1 methylation (Table 2), which suggests the absence of a general effect on CGI methylation. However, some gene-specific associations with anthropometric indices were identified (Table 2). Waist and hip circumference correlated positively with *WIF1* methylation*,* with stronger correlations in males; positively with *N33* methlaytion with stronger correlations in females; and negatively with LINE-1, with stronger correlations in females (Fig 2). FI was identified by the models for *APC* and *SFRP2* methylation and showed small positive correlations in males and negative correlations in females.

Participant height * sex interactions were selected in the models of methylation for *ESR1* and LINE-1 (Table 2). In both cases, the correlation was stronger in females. For *ESR1*, height correlated positively in females, *r* = 0.223 (unadjusted *P* < 0.5), but negatively in males, *r* = −0.179. Converse relationships were found for LINE-1, with a negative correlation in females, *r* = −0.260 (unadjusted *P* < 0.01) and positive correlation in males, *r* = 0.125 (Fig 2).

## White cell counts

Although not selected in the model of PCA1 methylation, either monocyte and/or WBC counts were selected in models for *WIF1*, *HPP1*, *SFRP1* and *SOX17* methylation. Monocyte count tended to be positively correlated with methylation, particularly in males and white cell count showed weak negative correlations with methlyation (Fig 2).

## Discussion

In this investigation, we quantified relationships between DNA methylation in the rectal mucosa of healthy subjects and biological factors implicated in epidemiologic studies as CRC risk factors. We adopted a modelling approach based on genetic algorithms to select variables that are the strongest predictors of gene-specific methylation. Although the levels of methylation at some of the loci we investigated were relatively low, we validated the robustness of these predictive models and assessed their ability to generalize to other data sets. Our observations confirm the importance of age as a major determinant of CGI methylation for most of the genes investigated, but also strongly suggest that folate status, and other nutritional and metabolic factors, modulate the methylation of specific genes. Thus, these factors contribute to the patterns of methylation acquired across the mucosa, and hence may play a role in the aetiology of CRC.

The single most important CRC risk factor is advancing age, with risk increasing exponentially beyond 50 years (Armitage & Doll, 1954). Previous studies have shown the concomitant methylation of multiple genes during aging, leading to the classification of this subset of genes as ‘Type A’, as distinct from ‘Type C’ genes, where methylation is thought to be cancer specific (Toyota *et al*., 1999). However, more recent studies argue against such a rigid classification (Belshaw *et al*., 2008). The strong association between age and methylation of 8 CGIs observed here is consistent with previous studies (Belshaw *et al*., 2008). This increases our confidence in the modelling technique, and reinforces the hypothesis that time-dependent silencing of genes that play an important role in regulating tissue renewal and maintaining homeostasis within individual stem cells in the colorectal epithelium contributes to field-wide changes in mucosal vulnerability to neoplasia. However, *APC* methylation was not significantly correlated with age nor with the methylation of the other genes and was also not strongly associated with the exposure variables. In this context, it is interesting to note that CGI methylation has only a limited effect on the expression of APC in human colorectal tumours, and consequently the role of *APC* methylation in carcinogenesis remains unclear (Segditsas *et al*., 2008).

The mechanisms leading to age-dependent CGI methylation are unclear. Recent studies showed that susceptible CGI in several cell types are enriched significantly for targets of polycomb group proteins, which are marked with both active and repressive histone modifications in embryonic stem cells (Teschendorff *et al*., 2010). This suggests that these CGI are more prone to *de novo* methylation. Expression of the DNA methyltransferase (DNMT) enzymes responsible for CpG methylation is modulated during aging in other tissues (Xiao *et al*., 2008). Increased expression of DNMT3B, a component of the polycomb repressor complex, was associated with sequential DNA methylation changes and colorectal neoplastic progression (Ibrahim *et al*., 2011), while overexpression of DNMT3b in the murine colon led to CGI methylation of genes including those shown here to be methylated with age (Steine *et al*., 2011).

Despite the strong influence of age on DNA methylation, our study shows considerable interindividual variation in the extent of age-related methylation for the genes investigated here. Previous twin studies have suggested that environmental factors contribute to this variance in age-related methylation for the majority of affected loci, while for a small subset of genes, genetic factors were more important (Bell *et al*., 2012; Talens *et al*., 2012). However, there is currently no evidence linking genetic factors to the variance in methylation of this panel of genes and we show that CGI and global DNA (LINE-1) methylation were associated with nutritional status and anthropometric measures, in a manner generally consistent with their impact on CRC risk as shown by epidemiologic studies.

We identified gender-related differences in the rate of age-related CGI methylation such that older males had higher levels of CGI methylation than females of the same age. In addition, for some individual genes, the strength of association between DNA methylation and height and waist circumference differed in males and females (Table 2). This is consistent with the higher overall risk of CRC in males compared with females (Koo & Leong, 2010).

There was an association between increased folate status, determined as either plasma or RCF, and increased CGI methylation, with significant positive correlations for *MYOD1*, *SFRP1*, *SFRP2*, *WIF1 and HPP1* and for the summary statistic for overall CGI methylation (PCA1). This is consistent with observations showing a positive association between RCF and methylation of *SFRP1* and *ESR1* in the apparently normal mucosa of previous polyp patients (Wallace *et al*., 2010). Folate plays a crucial role as a methyl group donor, contributing to the cellular supply of *S*-adenosylmethionine (SAM), the proximate source of methyl groups for DNA methylation. Indeed mathematical modelling of one-carbon metabolism supports the possibility that increased availability of folate might ‘drive’ DNA methylation through an increased intracellular SAM supply (Nijhout *et al*., 2006).

Concerns have been expressed that excessive intakes of folate through mandatory food fortification with folic acid may promote the development of CRC (Mathers, 2009) through various mechanisms including hypermethylation and silencing of tumour suppressor (and other genome defence) genes. However, epidemiological studies of the relationship between folate intake and risk of adenoma recurrence or CRC are inconsistent (Carroll *et al*., 2010; Fife *et al*., 2011; Kennedy *et al*., 2011) and there is a similar lack of agreement among studies investigating the relationship between PF status and risk of CRC. For example, Martinez *et al*. observed a reduced risk of adenoma recurrence in nonusers of multivitamin supplements in the highest quartile of folate status compared with those in the lowest quartile (Martinez *et al*., 2006), whereas in the Northern Sweden Health and Disease Cohort, higher PF levels were associated with greater CRC risk. Eussen *et al*. reported no association between PF and risk of CRC (Eussen *et al*., 2010), but recently Lee *et al*. reported lower CRC risk with lower PF status among participants in three large prospective trials in the USA (Lee *et al*., 2012). In view of these uncertainties, further studies are urgently required to understand the relationship between folate status, DNA methylation and colorectal carcinogenesis.

Vitamin B-12 and homocysteine are also important factors in one-carbon metabolism. Vitamin B-12 is a cofactor for methionine synthase responsible for the remethylation of homocysteine to methionine, while homocysteine is produced from *S*-adenosylhomocysteine, the coproduct of methylation reactions and a potent inhibitor of DNA methylation. Al-Ghnaniem *et al*. reported a negative association between vitamin B-12 and methylation of *ESR1* CGI in the normal colonic mucosa, while a positive association was observed for homocysteine (Al-Ghnaniem *et al*., 2007). In contrast, there was a negative association between plasma homocysteine and global DNA methylation in colon tissue (Pufulete *et al*., 2005). In this study, we found no associations with either vitamin B-12 or homocysteine and DNA methylation.

Vitamin D status was inversely related to CGI methylation in this set of genes, which is consistent with the convincing evidence from epidemiological studies for a protective effect of vitamin D against CRC risk (Lee *et al*., 2011). Putative mechanisms for this protective effect include inhibition of cell proliferation and promotion of cell differentiation by antagonism of the Wnt signalling pathway, or by induction of the DNA demethylation-dependent expression of E-cadherin (Palmer *et al*., 2001; Lopes *et al*., 2012).

Epidemiological evidence suggests a protective effect of selenium against CRC. Selenium modulates DNA methylation in both CRC cell lines and in colon tissue of rats by inhibition of DNMT expression (Davis *et al*., 2000) and inhibits DNMT activity *in vitro* (Fiala *et al*., 1998), which suggest that selenium may decrease CRC risk by preventing aberrant DNA methylation. In this study, there was a weak negative correlation between selenium status and methylation of *WIF1* in both genders. For most of the genes studied, this weak negative correlation was found predominantly in females. In contrast, selenium status correlated positively with LINE-1 methylation in females. This suggests the possibility of a sex-dependent interaction between selenium, DNA methylation (at least for some genes and for LINE-1) and CRC risk. Sex-dependent effects of selenium on cancer risk have been reported for bladder and oesophageal cancers (Amaral *et al*., 2010; Steevens *et al*., 2010) and may reflect differential responses to selenium by gender (Combs *et al*., 2011).

Adult height is positively associated with CRC risk in women but not in men (Park *et al*., 2012). Height is influenced by many genetic and environmental exposures acting *in utero*, and during childhood. In agreement with the specific link between height and CRC risk, we observed that height was correlated significantly with increased *ESR1* methylation*,* and with decreased LINE-1 methylation only in females.

Obesity *per se,* and increased central adiposity in particular, are significantly associated with increased CRC risk (Pischon *et al*., 2006). Although the mechanisms remain to be established, increased low-grade, systemic inflammatory signals produced by increased adipose tissue may contribute to greater cancer risk (Johnson & Lund, 2007). Higher waist and hip circumference were associated significantly with higher methylation of several CGIs in this study, and with lower methylation of LINE-1. Further support for an association between systemic inflammation and CGI methylation is suggested by the significant positive association between aberrant DNA methylation and blood monocyte count. This subset of WBCs is elevated in response to infection and chronic inflammatory conditions such as ulcerative colitis (Mee *et al*., 1980). However, no participants in the BORICC (Biomarkers of Risk of Colorectal Cancer) study showed any evidence of inflammatory bowel disease.

In conclusion, the application of a multivariate modelling approach to quantitative methylation analyses of DNA from the normal rectal mucosa of healthy subjects confirmed the importance of age as a major determinant of methylation but also identified significant relationships between DNA methylation and several nutritional, metabolic and anthropometric factors related to CRC. Our panel of genes consisted mainly of genes involved in the Wnt signalling pathway, which plays a central role in controlling tissue renewal in the colon and is overactive in the vast majority of colorectal tumours (Gregorieff & Clevers, 2005). Our observations suggest that regulation of this pathway is susceptible to modulation by the combined effects of aging, nutrition and metabolic status, which would have important implications for the maintenance of colorectal tissue homeostasis. These relationships provide a biologically plausible mechanism for the influence of environmental factors on CRC risk, which requires further investigation. Future studies, utilizing this approach and incorporating more environmental and metabolic factors, will further our understanding of how the environment modulates the epigenomes of normal cells, and the consequential influence on CRC risk.

## Experimental procedures

### Subject recruitment and sample collection

Participants in the BORICC study were recruited from patients attending Wansbeck General Hospital, Northumberland, UK for diagnostic endoscopy, who had no known familial predisposition to colorectal cancer, and who were shown by flexible sigmoidoscopy to be free from colorectal neoplasia or inflammatory disease. While this procedure is considered sufficient for the clinical diagnosis of absence of disease, there remains a small possibility that conditions affecting the proximal colon may be present. Patients were invited to participate in the study and written informed consent was obtained. Ethical approval for the study was obtained from the Northumberland Local Research Ethics Committee (Project reference NLREC2/2001). Nine rectal mucosal samples were taken 10 cm from the anal verge by pinch biopsy from 185 volunteers (84 males, 101 females; median age 50 year, range 17–77 year). Blood was collected by venepuncture, and samples of whole blood, serum and plasma were prepared, flash-frozen with liquid nitrogen and stored at −80°C. Anthropometric measurements (height, weight and waist and hip circumferences) were made and Fatness Index (FI) was calculated (see Supplementary Methods).

### DNA methylation analysis

Biopsies were stored at −80°C before analysis. Genomic DNA was extracted and purified using a Genelute DNA extraction kit (Sigma-Aldrich, Gillingham, UK). CGI methylation analysis was carried out using a quantitative methylation-specific PCR assay described previously (Belshaw *et al*., 2008), applied to the following genes: *SFRP1*, *SFRP2*, *AXIN2*, *WIF1*, *APC*, *HPP1/TMEFF2*, *DKK1*, *SOX17*, *ESR1*, *MYOD1* and *N33*. PCR primer sequences are provided in Supplementary Table S4. Methylation of LINE-1 was measured as an index of global DNA methylation using the qPCR approach (Iacopetta *et al*., 2007).

### Nutritional status

Blood samples were analysed for RCF, PF, vitamin B12, WBCs and monocytes using standard clinical laboratory assays. Plasma homocysteine was measured using reverse-phase HPLC as described by Loehrer *et al*. (Loehrer *et al*., 1996). Plasma 25-Hydroxyvitamin D was determined using a commercial enzyme immunoassay kit (Immunodiagnostics Systems Limited, Tyne & Wear, UK). Plasma Selenium concentration was measured by ICP MS.

### Statistical analysis

Data were analysed using Matlab (Mathworks Inc., Cambridge, UK). Pearson correlation was used to investigate relationships of CGI methylation with each independent variable (age, nutritional status and anthropometric indices) and to aid interpretation of the subset regression models (see below).

Data were available for 185 subjects for all variables except for methylation of *ESR1*, *MYOD* and *N33* where data were available for 174 subjects only due to failure of DNA amplification for some subjects. To facilitate equal weighting for each gene in subsequent analyses, methylation values (expressed initially as%) were auto-scaled to span the range [0.01–1]. The first principal component score vector PCA1, based on all nine genes, was calculated as an overall indicator of CGI methylation status for 174 subjects and was also auto-scaled.

We applied multivariate modelling to predict methylation levels using an approach that selected a subset of explanatory variables, while catering for potential gender differences in each selected cofactor. Each selected variable could be associated with a single ‘term’ or have separate terms for men and women. ANCOVA models of gene methylation were built from predictors comprising 1 categorical variable, ‘Sex’; and 16 continuous variables (anthropometric and blood measurements) using GA (Tapp *et al*., 2003; Kemsley *et al*., 2007). For further information on the GA-ANCOVA modelling, see Supplementary Methods.
