# Supplementary material for: Nutritional factors and gender influence age-related DNA methylation in the human rectal mucosa
Source: Aging Cell. 2012 Dec 6;12(1):148–55. doi: 10.1111/acel.12030 (PMC3572581; doi:10.1111/acel.12030)
Supplement: Supplementary file 1 [file acel0012-0148-sd1.doc]

**Supplementary methods**

***Estimated body fat***

The method of Watson et al. was used to estimate total body water for all subjects. These data combined with Siri’s values for the hydration of fat-free tissue (0.72), and the density of water at body temperature, were used to calculate the Fatness Index (FI), an anthropometric estimate of percentage body fat for each volunteer.

***GA-ANCOVA modelling***

*Chromosomes*, which describe candidate models, comprised 33 element binary vectors representing combinations of main effects and sex-interaction terms. Particular to this GA application was a faulty *repair* stage: if absent, main effects corresponding to the interaction terms were inserted (*repaired*), but with a 4% chance of a *fault* subsequently removing all interactions and the ‘Sex’ main effect. This mechanism controlled the proliferation of interaction terms. Population fitness was assessed from the mean Q^2^ statistic over 5 rounds of 10-block single cross-validation . Block membership was permuted after each round. The population size was 40; the initial population contained 8 (pre-repair) terms per chromosome; the fittest 50% were eligible to breed and the chromosomes within each new population (*generation)* were genetically unique. A single implementation of the GA, termed an *epoch*, ended either when one model performed best over 5 successive generations; or when a maximum of 50 generations was reached, in which case the most frequently occurring ‘best’ model was chosen (or best models in the case of ties). Model consistency was assessed over 10 epochs.

In order to assess over-fitting, the ability of each model to generalise to other data sets was estimated from 10 rounds of 10-block double cross-validation (2CV) . The proportion of explained variance was estimated using Fisher’s z-transformation to average the correlations of the 2CV predictions. Models failed under 2CV when: *r*_2CV_ < tanh(1.96/√(*n_i_* – 3)), where *r*_2CV_ is the averaged correlation and *n_i_* is the number of subjects in the dataset.

**Supplementary references**

1. Watson PE, Watson ID, & Batt RD (1980) Total-Body Water Volumes for Adult Males and Females Estimated from Simple Anthropometric Measurements. *American Journal of Clinical Nutrition* 33(1):27-39.

2. Siri WE (1956) The gross composition of the body. *Adv Biol Med Phys* 4:239-280.

3. Westerhuis JA, van Velzen EJJ, Hoefsloot HCJ, & Smilde AK (2008) Discriminant Q(2) (DQ(2)) for improved discrimination in PLSDA models. *Metabolomics* 4(4):293-296.

4. Westerhuis JA, et al. (2008) Assessment of PLSDA cross validation. *Metabolomics* 4(1):81-89.
