# Supplementary material for: Nutritional factors and gender influence age-related DNA methylation in the human rectal mucosa
Source: Aging Cell. 2012 Dec 6;12(1):148–55. doi: 10.1111/acel.12030 (PMC3572581; doi:10.1111/acel.12030)
Supplement: Supplementary file 2 [file acel0012-0148-sd2.doc]

**GA-ANCOVA models**

**1.1 Abbreviations**

| Quantity | Abbreviation in tables | Abbreviation in figures |
| --- | --- | --- |
| Age | Age | Age |
| Sex | Sex | Sex |
| Height | Height | Ht |
| Weight | - | - |
| Body mass index | BMI | BMI |
| Waist circumference | Waist | Waist |
| Hip circumference | Hip | Hip |
| Waste to hip ratio | WHR | WHR |
| Plasma folate | SerumFol | SFol |
| Red cell folate | RedCFol | RCFol |
| Homocysteine | - | - |
| White cell count | WhiteCells | WBC |
| Monocytes count | Moncyt | Mon |
| Vitamin D | vitD | vitD |
| Selenium | Selenium | Se |
| Fatness index | FI | FI |
| Vitamin B12 | B12 | B12 |

**1.2 Interpretation of GA-ANCOVA models**

For each of the models the following summaries are provided

1. ANCOVA tables calculated using Matlabs ‘hierarchical’ sum of squares (equivalent to R’s Type II sum of squares).
2. Regression coefficients multiplied by standard deviation of the corresponding cofactor based on whole group data. This allows the relative sizes of the regression coefficients to be compared.
3. TinyLVR loading plots (Tapp et al 2011, Chemom Intell Lab Syst; **105**: 19–26) based on unit variance scaled data, again using the standard deviation of the whole group (men and women) data. Where no sex-interaction terms are present, the **w**_1_ loadings (plotted on the x-axis) are proportionate to the Pearson correlation between the methylation levels and a particular cofactor. When there are sex interactions, the **w**_1_ coefficients are almost proportionate to the correlation. The loading plots also include a second set of axis that have been rotated in a clockwise direction. Projections of the loadings onto the solid rotated axis are proportional to the regression coefficients. When there is little systematic variation unrelated to the quantity being predicted, then the regression coefficients are nearly proportional to the corresponding correlation values and therefore the secondary axis is only slightly rotated. The angle of rotations indicated the extent that X-specific systematic variation is incorporated into the model. The loading plot gives a visual link between univariate quantities (correlation) and the multivariate regression model. In certain regions of the plot, ‘flip-zones’, the sign of the regression coefficient will differs from the correlation between the cofactor and dependant variable.
4. Scatterplots of methylation against subject age including actual values and not cross-validated predictions and differentiated by gender.

**2.1 LINE-1**

| Source | Sum Sq. | d.f. | Mean Sq. | F | Prob>F |
| --- | --- | --- | --- | --- | --- |
| Sex | 0.0096 | 1 | 0.0096 | 0.47 | 0.4948 |
| Height | 0.0131 | 1 | 0.0131 | 0.64 | 0.4250 |
| BMI | 0.0717 | 1 | 0.0717 | 3.50 | 0.0630 |
| Hip | 0.1514 | 1 | 0.1514 | 7.40 | 0.0072 |
| VitD | 0.0298 | 1 | 0.0298 | 1.45 | 0.2294 |
| Selenium | 0.0818 | 1 | 0.0818 | 3.99 | 0.0472 |
| B12 | 0.0592 | 1 | 0.0592 | 2.89 | 0.0906 |
| Sex*Height | 0.1463 | 1 | 0.1463 | 7.15 | 0.0082 |
| Error | 3.6024 | 176 | 0.0205 |  |  |
| Total | 4.1969 | 184 |  |  |  |

LINE-1 Type II ANCOVA table of GA selected model

| Gene | Gender | Height | BMI | Hip | VitD | Selenium | B12 |
| --- | --- | --- | --- | --- | --- | --- | --- |
| LINE-1 | Men | 0.0519 | 0.0521 | -0.0781 | 0.0136 | 0.0235 | -0.0193 |
|  | Women | -0.0403 | 0.0521 | -0.0781 | 0.0136 | 0.0235 | -0.0193 |

LINE-1: Regression coefficients of continuous variables multiplied by their population standard deviations.

| 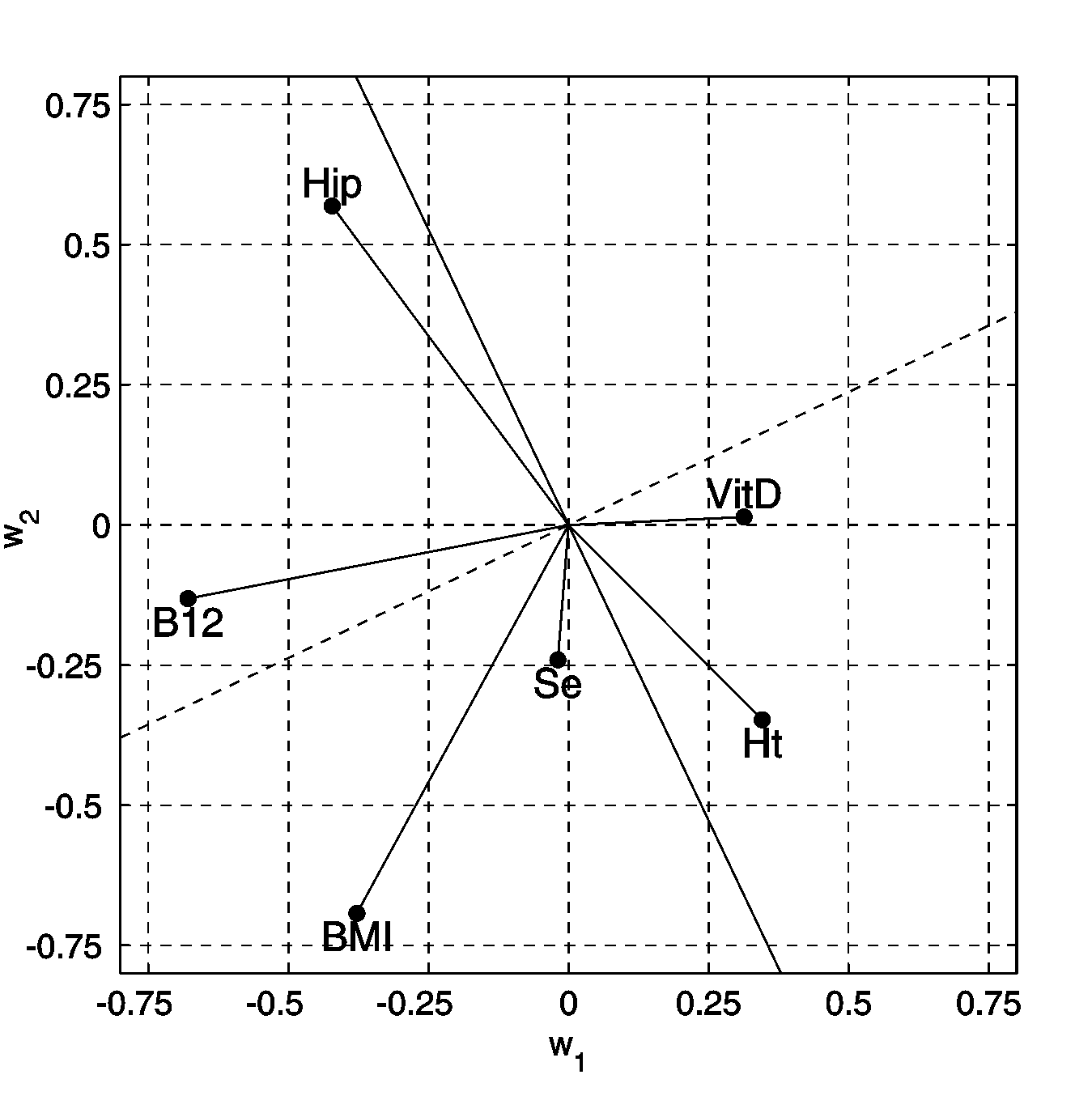 | 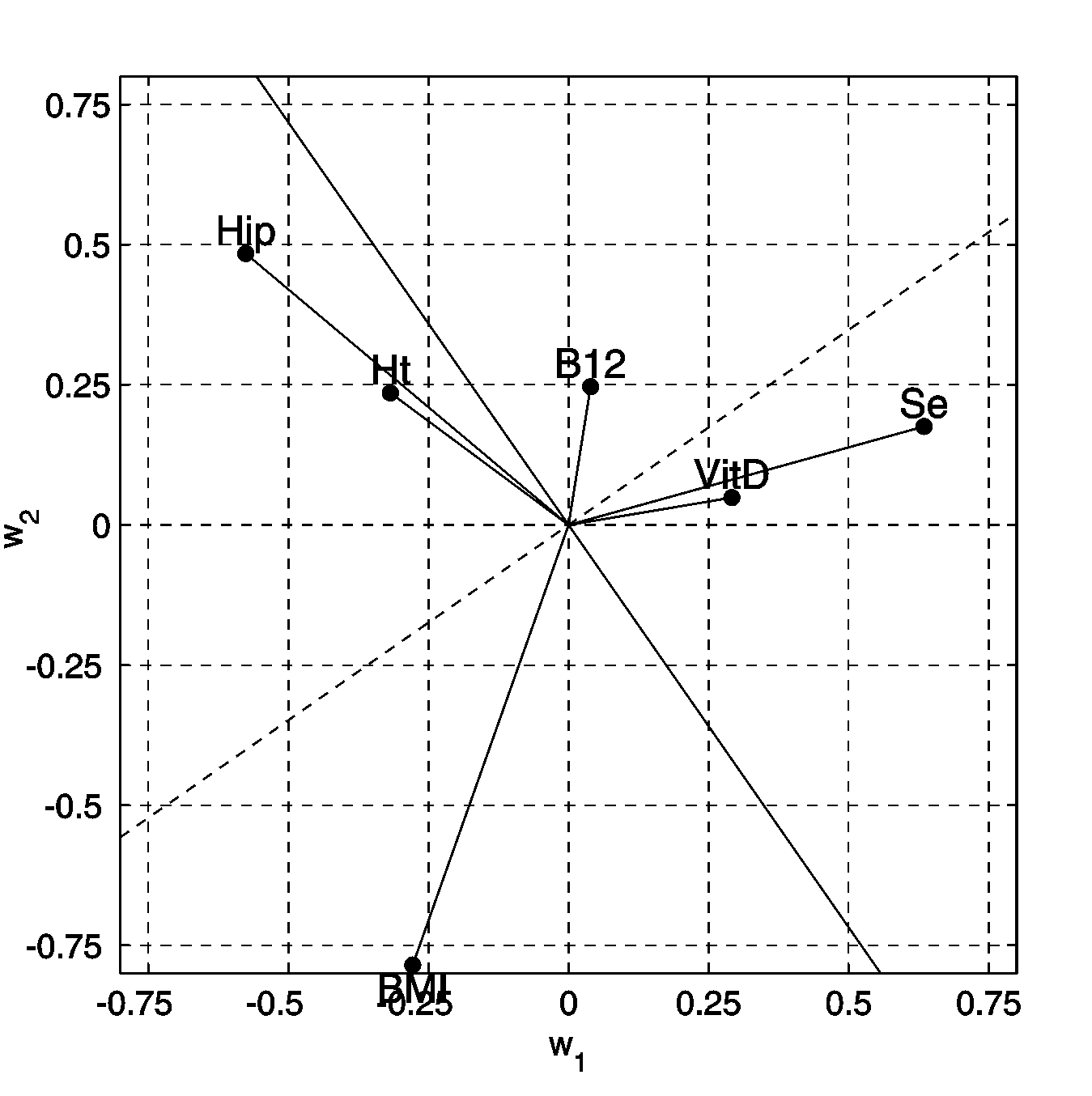 |
| --- | --- |
| Men: TinyLVR loading plot | Women: TinyLVR loading plot |

| **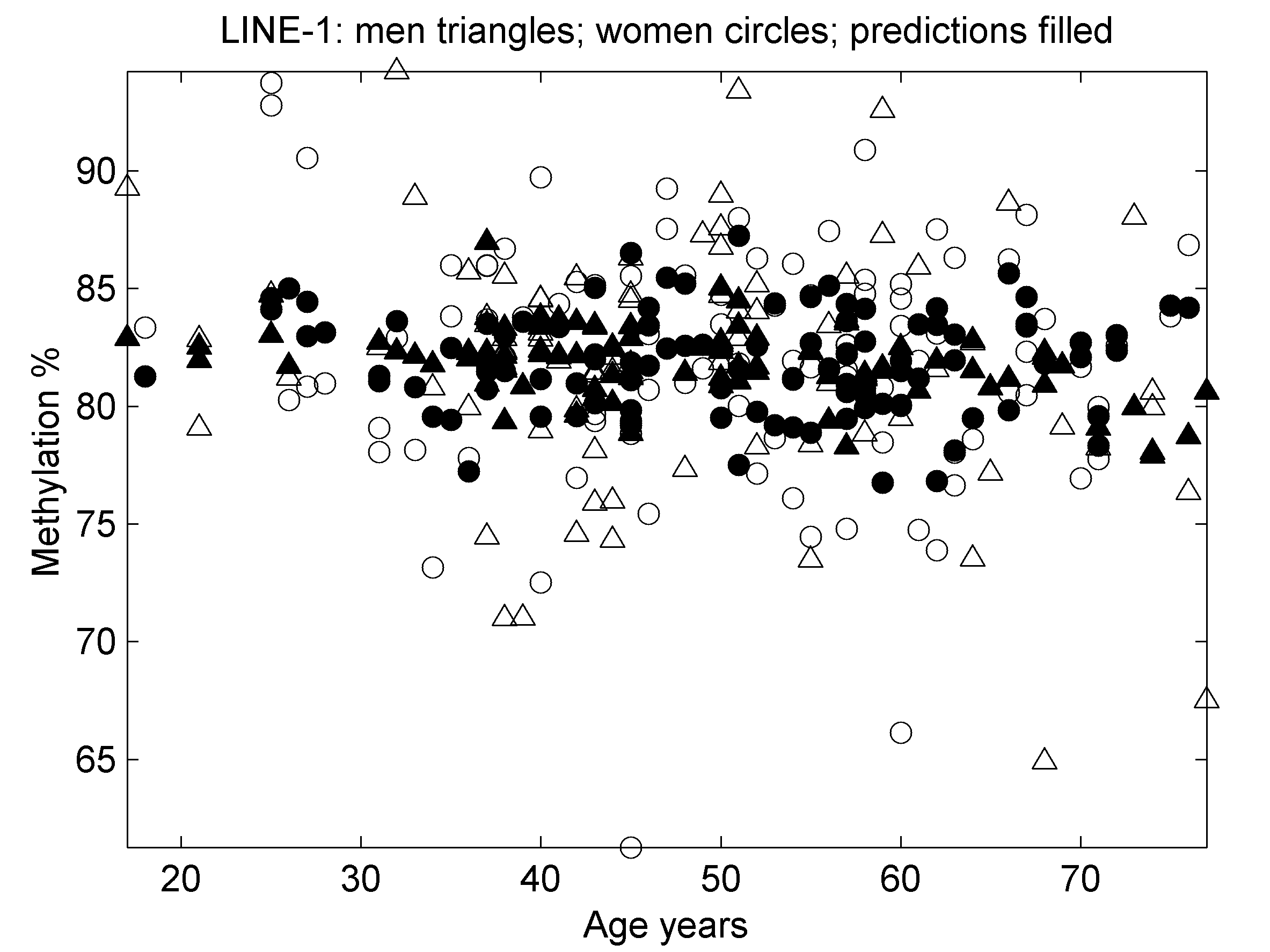** |
| --- |
| Variation in methylation with age: actual unscaled values and non-CV predictions |

**2.2 *HPP1***

| Source | Sum Sq. | d.f. | Mean Sq. | F | Prob>F |
| --- | --- | --- | --- | --- | --- |
| Age | 1.0412 | 1 | 1.0412 | 30.81 | 1.01E-07 |
| Sex | 0.0208 | 1 | 0.0208 | 0.62 | 0.4334 |
| Moncyt | 0.0070 | 1 | 0.0070 | 0.21 | 0.6490 |
| Age*Sex | 0.1176 | 1 | 0.1176 | 3.48 | 0.0638 |
| Sex*Moncyt | 0.2553 | 1 | 0.2553 | 7.56 | 0.0066 |
| Error | 6.0483 | 179 | 0.0338 |  |  |
| Total | 7.4598 | 184 |  |  |  |

HPP1 Type II ANCOVA table of GA selected model

| Gene | Gender | Age | Moncyt |
| --- | --- | --- | --- |
| HPP1 | Men | 0.1036 | 0.0499 |
|  | Women | 0.0523 | -0.0272 |

HPP1: Regression coefficients of continuous variables multiplied by their population standard deviations.

| 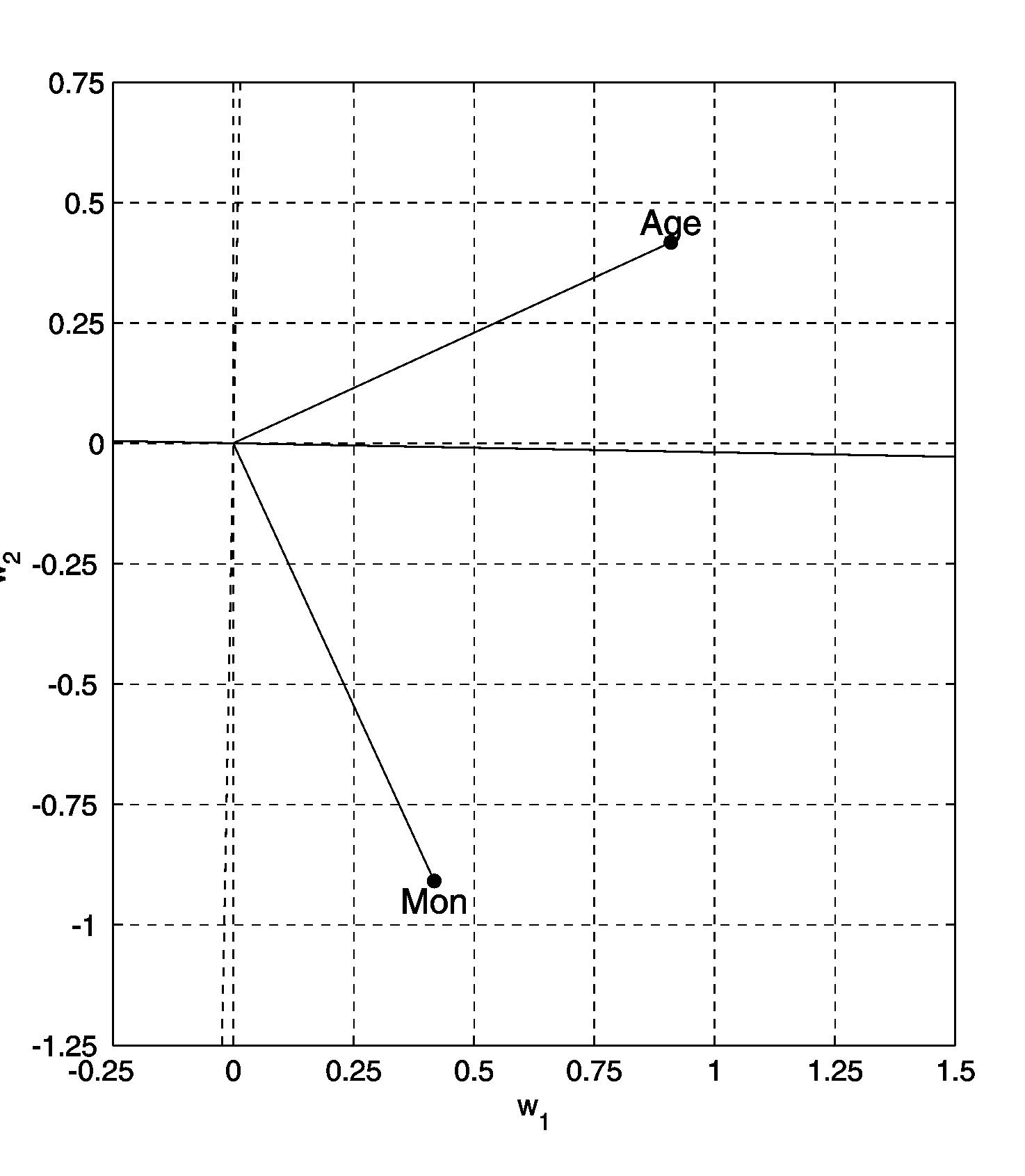 | 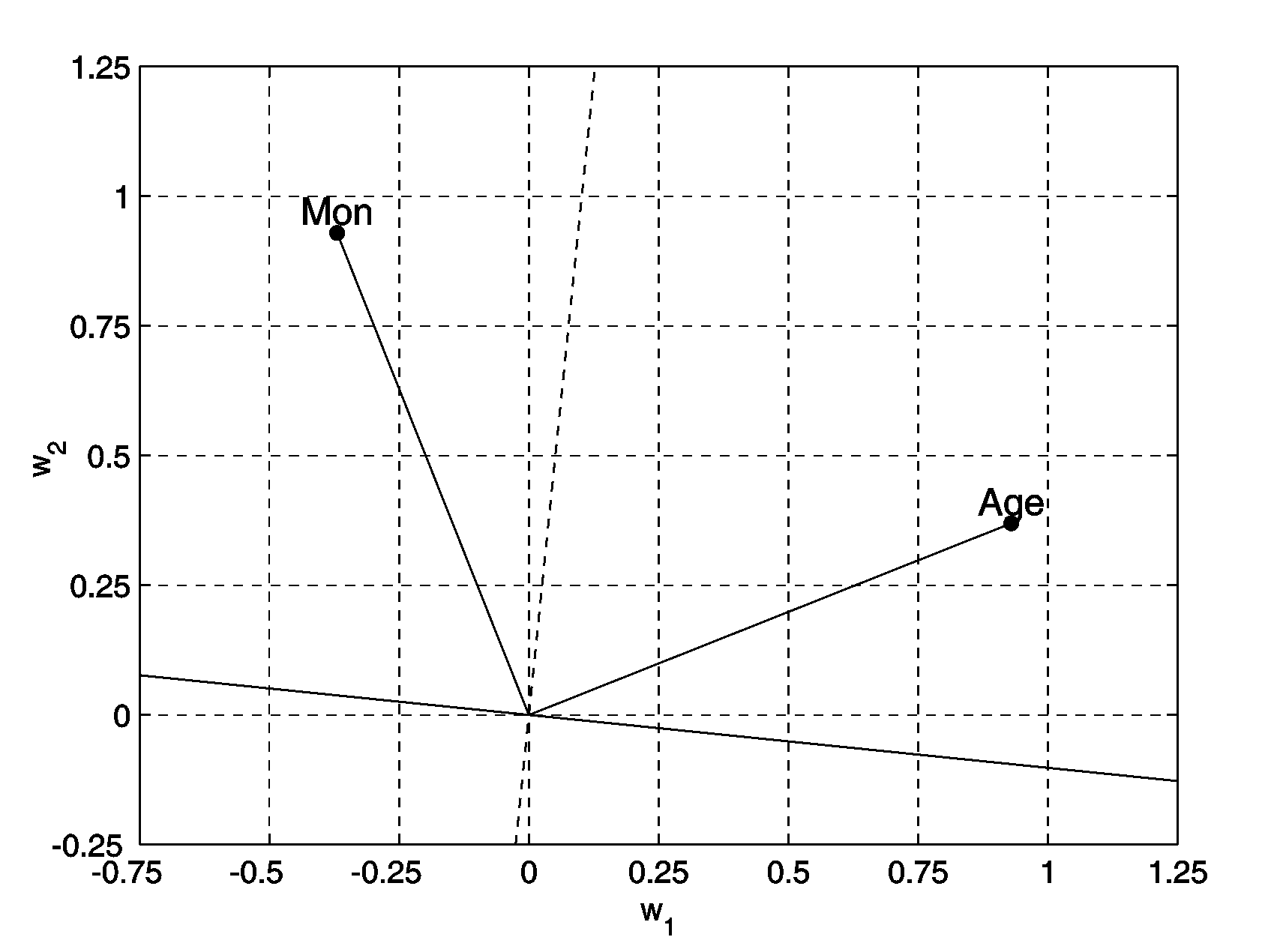 |
| --- | --- |
| Men: TinyLVR loading plot | Women: TinyLVR loading plot |

| **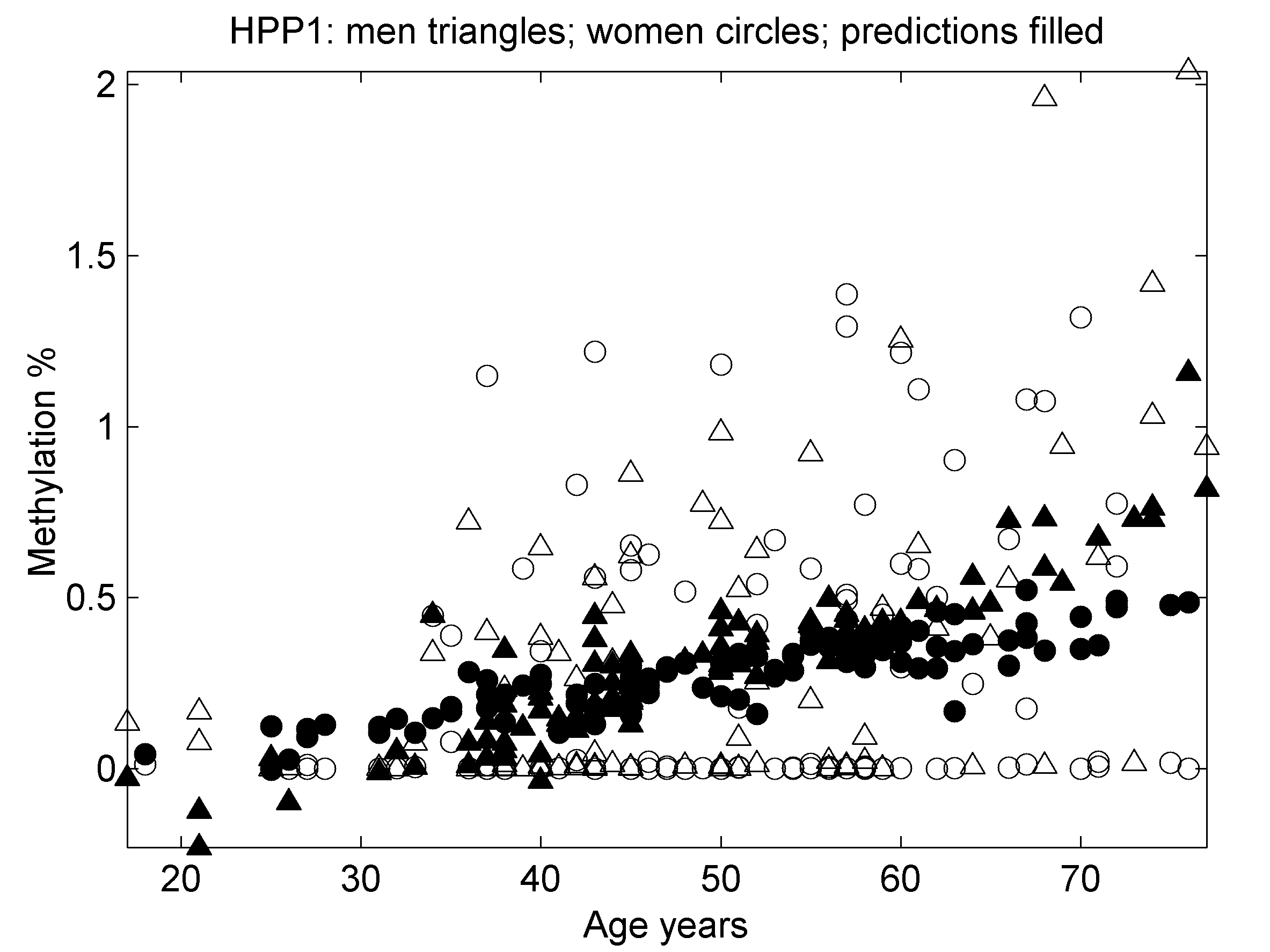** |
| --- |
| Variation in methylation with age: actual unscaled values and non-CV predictions |

**2.3 *APC***

| Source | Sum Sq. | d.f. | Mean Sq. | F | Prob>F |
| --- | --- | --- | --- | --- | --- |
| Age | 0.0427 | 1 | 0.0427 | 2.53 | 0.1132 |
| VitD | 0.0515 | 1 | 0.0515 | 3.05 | 0.0823 |
| FI | 0.0489 | 1 | 0.0489 | 2.90 | 0.0903 |
| Error | 3.0535 | 181 | 0.0169 |  |  |
| Total | 3.2065 | 184 |  |  |  |

APC Type II ANCOVA table of GA selected model

| Gene | Gender | Age | VitD | FI |
| --- | --- | --- | --- | --- |
| APC | Both | 0.0160 | -0.0171 | -0.0167 |

APC: Regression coefficients of continuous variables multiplied by their population standard deviations.

| 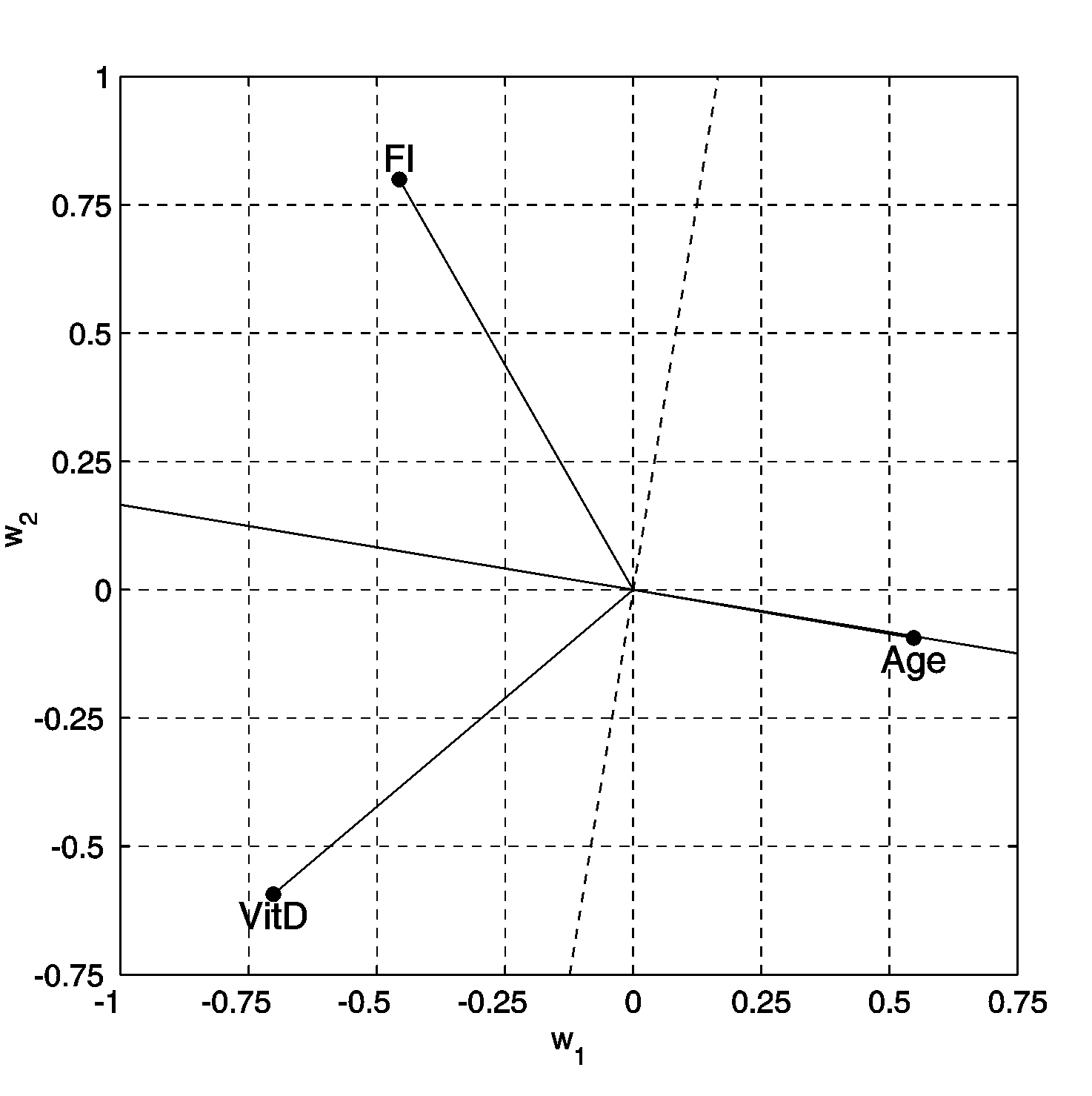 |
| --- |
| TinyLVR loading plot |

| **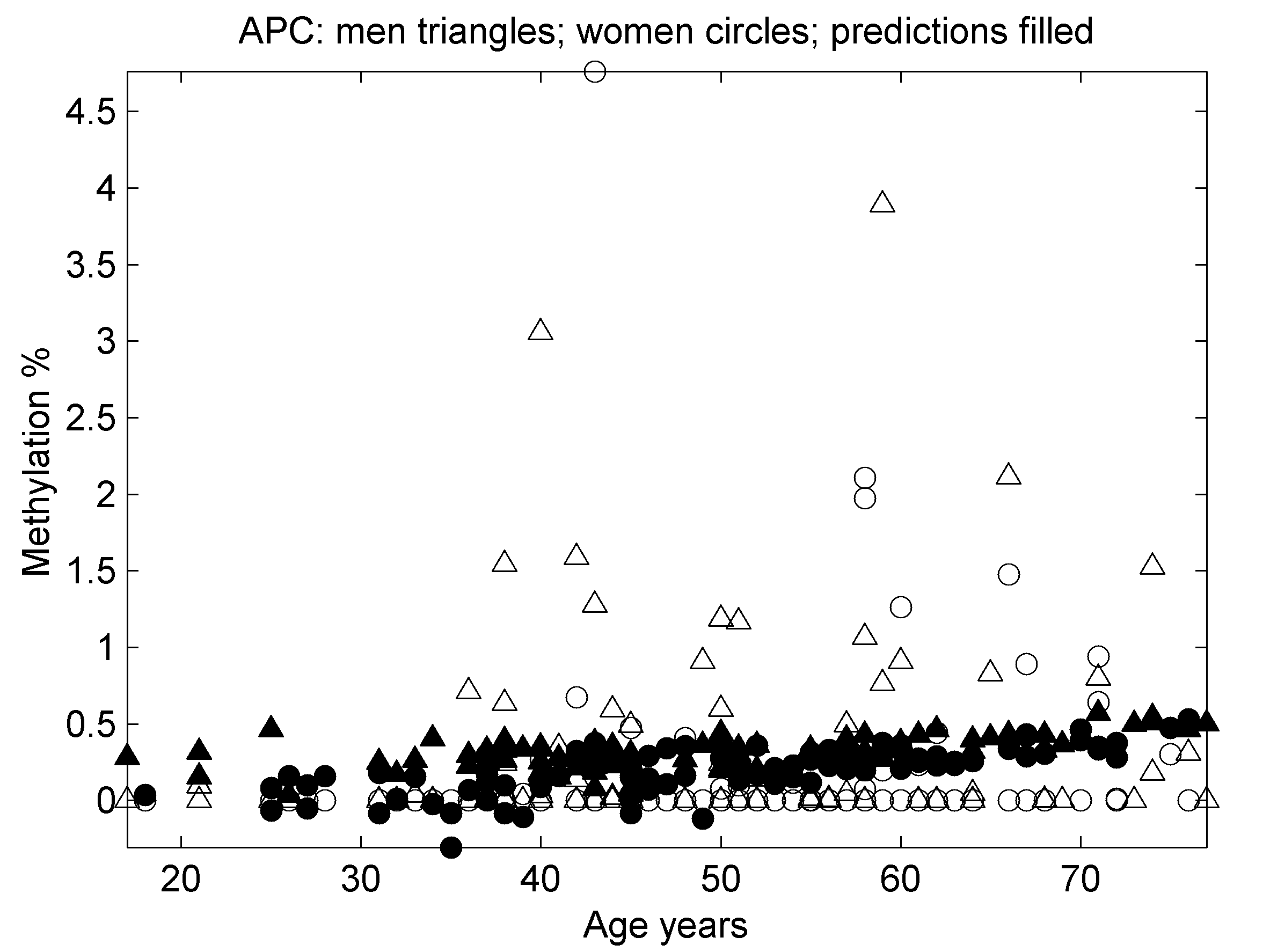** |
| --- |
| Variation in methylation with age: actual unscaled values and non-CV predictions |

**2.4 *SFRP1***

| Source | Sum Sq. | d.f. | Mean Sq. | F | Prob>F |
| --- | --- | --- | --- | --- | --- |
| Age | 0.1679 | 1 | 0.1679 | 10.95 | 0.0011 |
| Sex | 0.1086 | 1 | 0.1086 | 7.08 | 0.0085 |
| RedCFol | 0.0981 | 1 | 0.0981 | 6.40 | 0.0123 |
| Moncyt | 0.0391 | 1 | 0.0391 | 2.55 | 0.1120 |
| Sex*RedCFol | 0.1871 | 1 | 0.1871 | 12.21 | 0.0006 |
| Error | 2.7439 | 179 | 0.0153 |  |  |
| Total | 3.4185 | 184 |  |  |  |

SFRP1 Type II ANCOVA table of GA selected model

| Gene | Gender | Age | RedCFol | Moncyt |
| --- | --- | --- | --- | --- |
| SFRP1 | Men | 0.0310 | 0.0587 | 0.0150 |
|  | women | 0.0310 | -0.0060 | 0.0150 |

SFRP1: Regression coefficients of continuous variables multiplied by their population standard deviations.

| 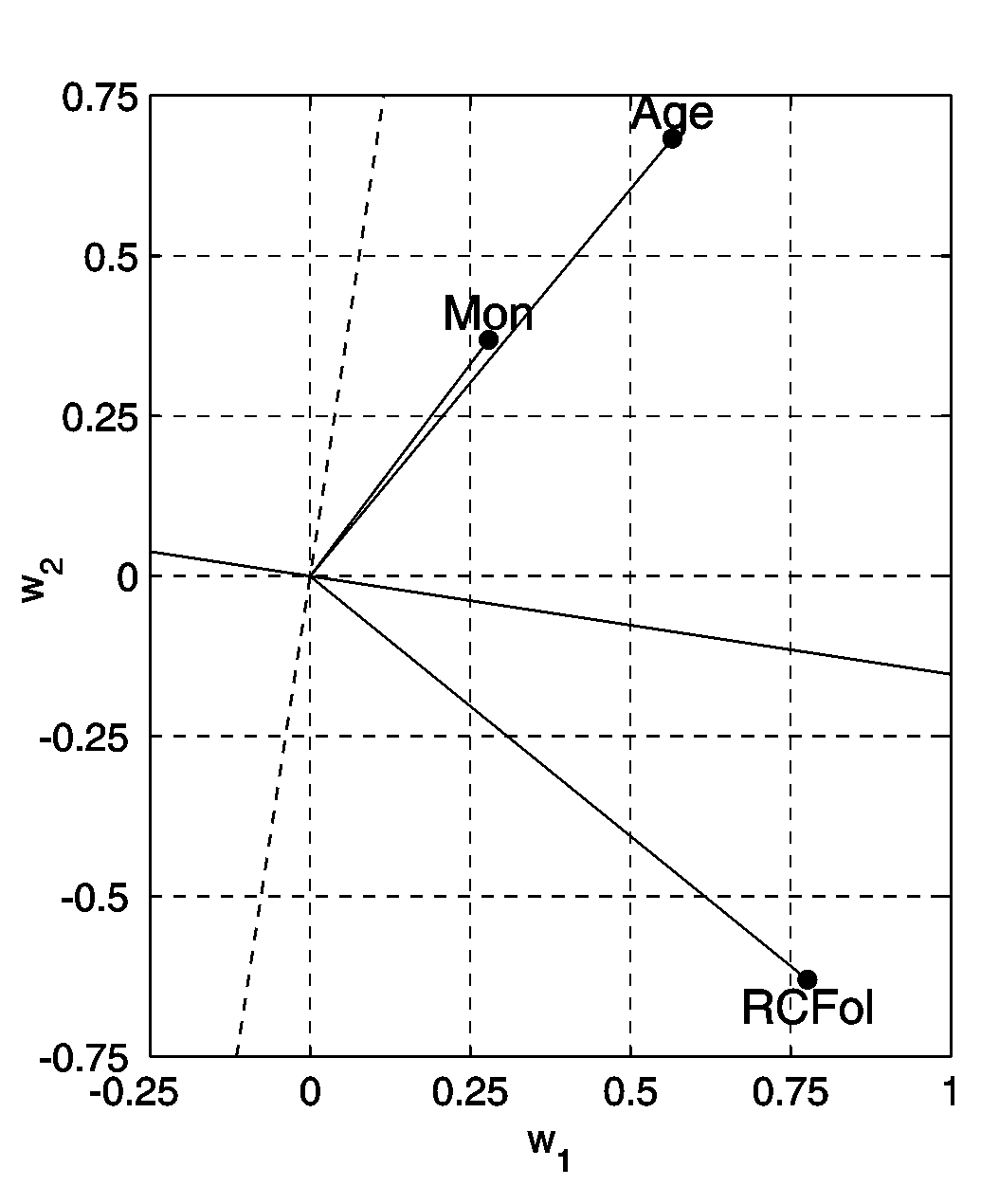 | 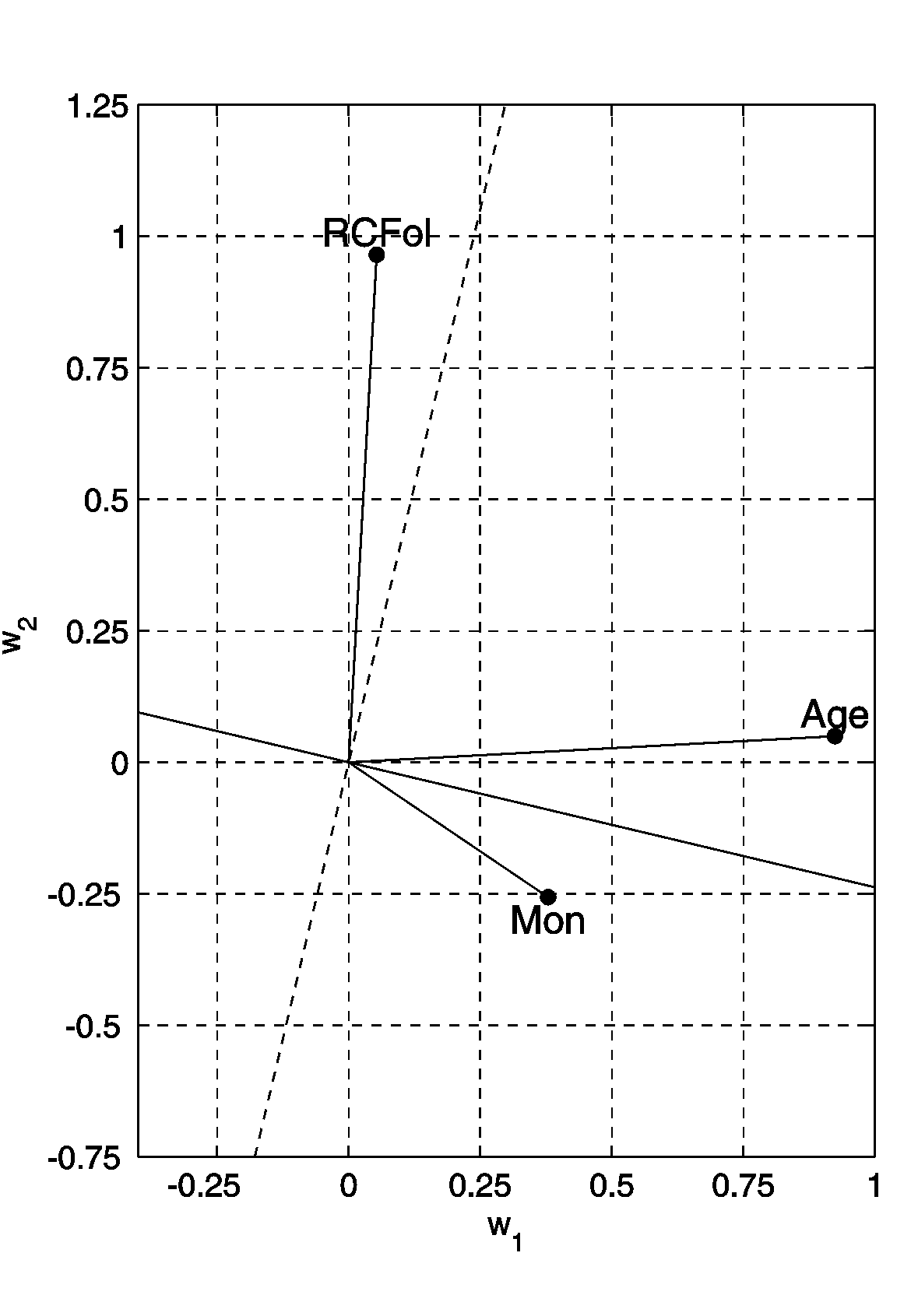 |
| --- | --- |
| Men: TinyLVR loading plot | Women: TinyLVR loading plot |

| **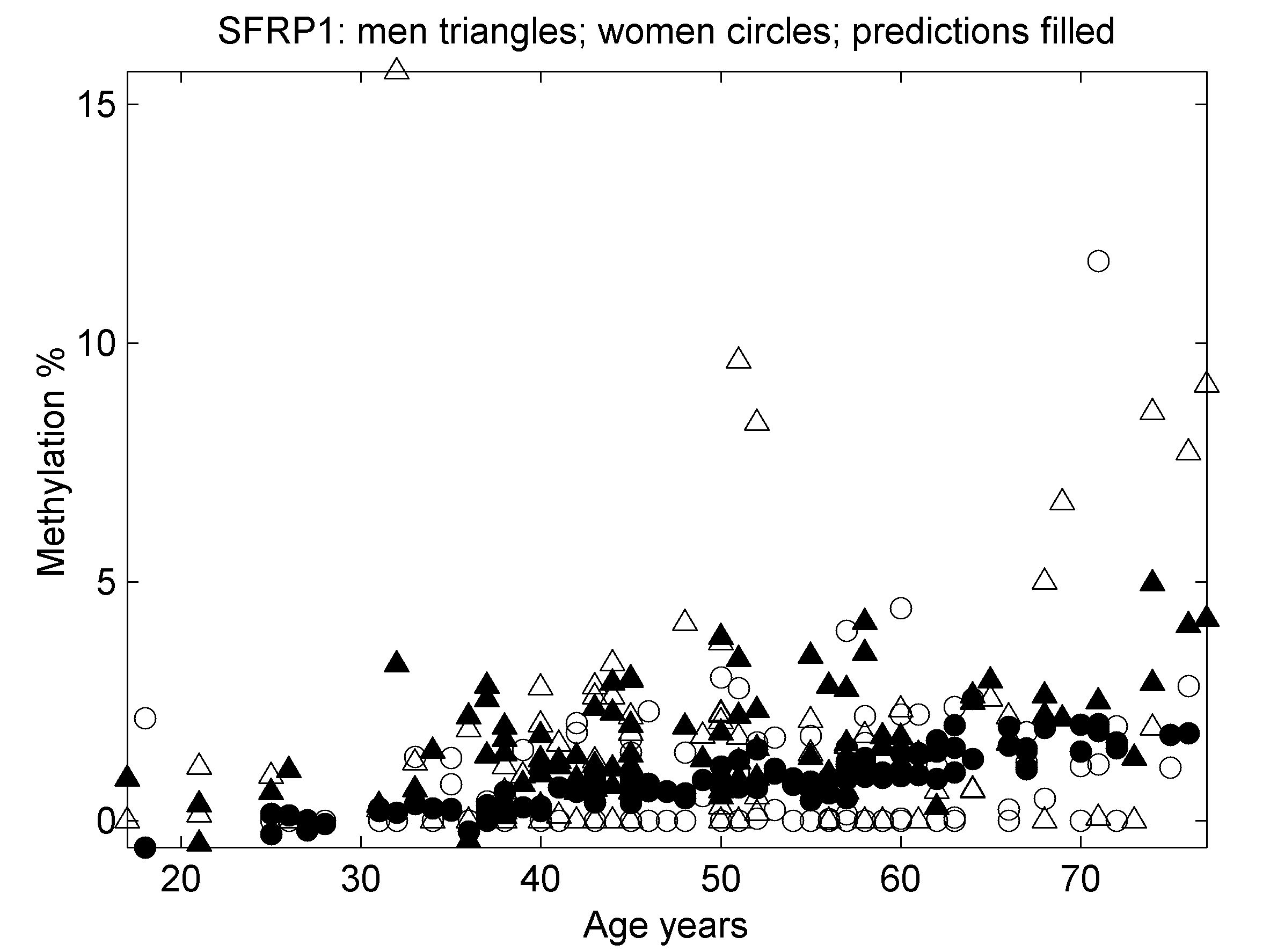** |
| --- |
| Variation in methylation with age: actual unscaled values and non-CV predictions |

**2.5 *SFRP2***

| Source | Sum Sq. | d.f. | Mean Sq. | F | Prob>F |
| --- | --- | --- | --- | --- | --- |
| Age | 0.4303 | 1 | 0.4303 | 20.36 | 1.15E-05 |
| FI | 0.0657 | 1 | 0.0657 | 3.11 | 0.0796 |
| Error | 3.8462 | 182 | 0.0211 |  |  |
| Total | 4.2908 | 184 |  |  |  |

SFRP2 Type II ANCOVA table of GA selected model

| Gene | Gender | Age | FI |
| --- | --- | --- | --- |
| SFRP2 | Both | 0.0495 | -0.0193 |

SFRP2: Regression coefficients of continuous variables multiplied by their population standard deviations.

| 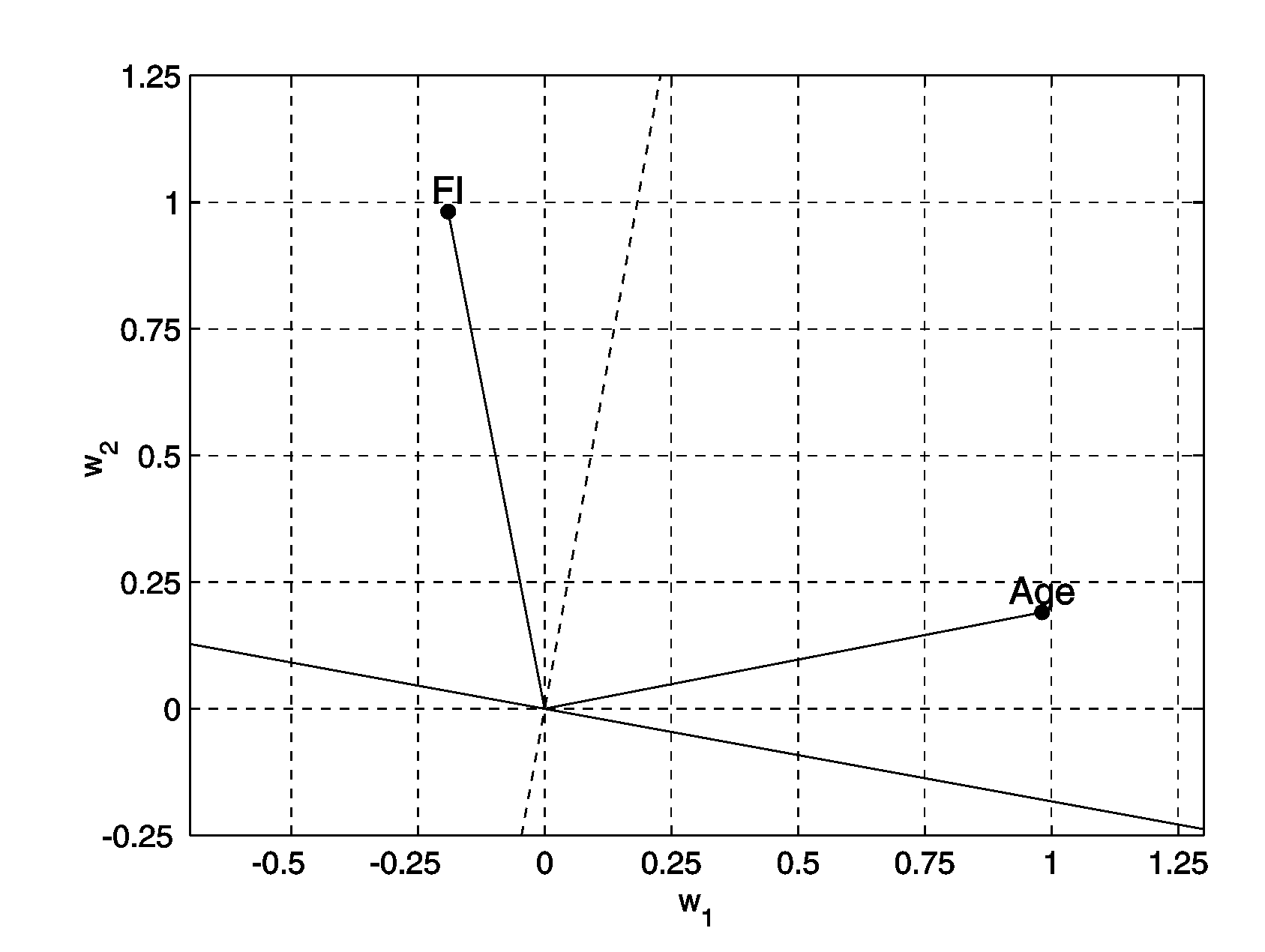 |
| --- |
| TinyLVR loading plot |

| **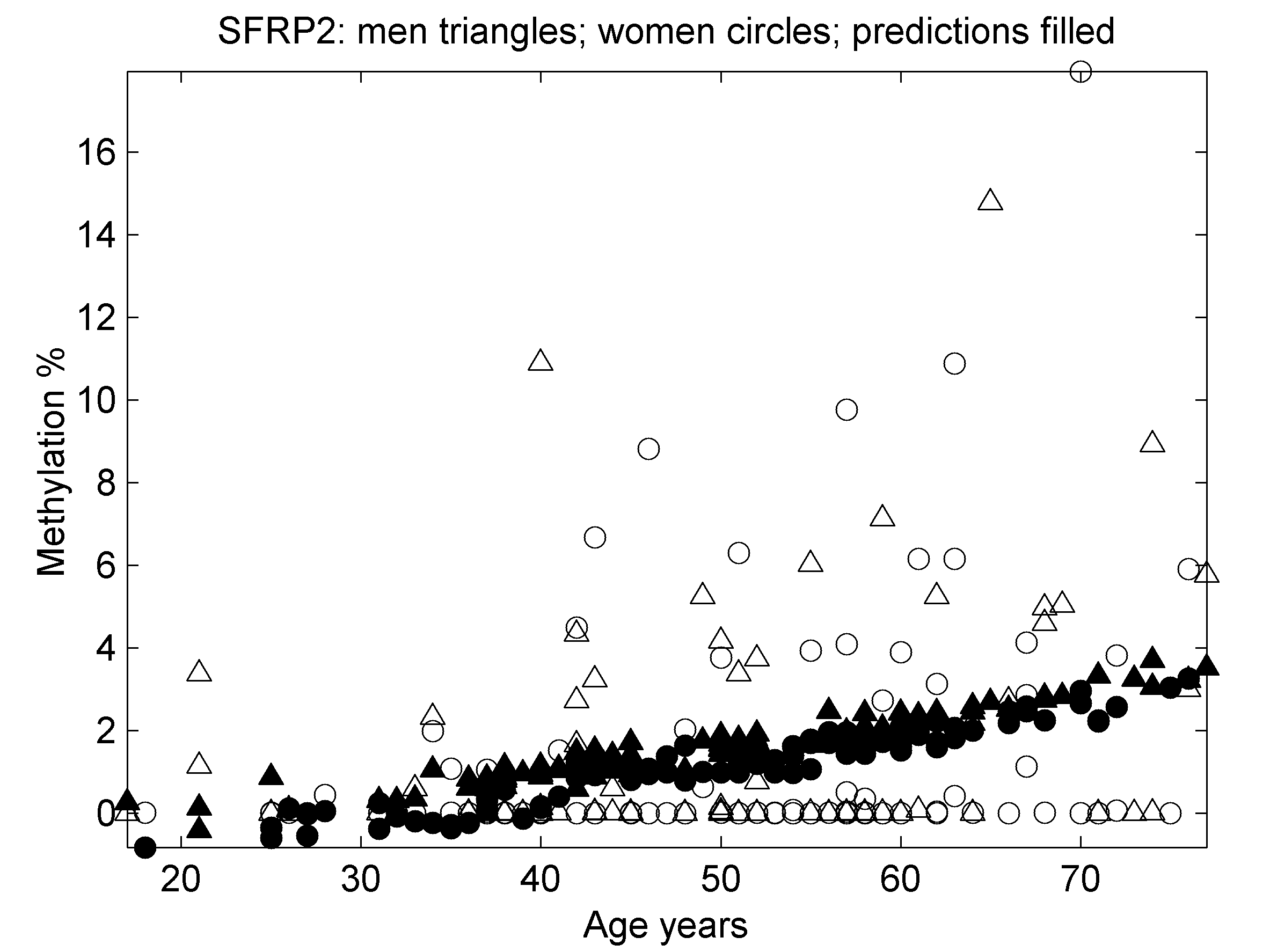** |
| --- |
| Variation in methylation with age: actual unscaled values and non-CV predictions |

**2.6 *SOX17***

| Source | Sum Sq. | d.f. | Mean Sq. | F | Prob>F |
| --- | --- | --- | --- | --- | --- |
| Age | 0.2875 | 1 | 0.2875 | 25.05 | 1.32E-06 |
| Sex | 0.03402 | 1 | 0.0340 | 2.96 | 0.0869 |
| WhiteCells | 0.0281 | 1 | 0.0281 | 2.45 | 0.1197 |
| Age*Sex | 0.0532 | 1 | 0.0532 | 4.63 | 0.0327 |
| Error | 2.0659 | 180 | 0.0115 |  |  |
| Total | 2.4787 | 184 |  |  |  |

SOX17 Type II ANCOVA table of GA selected model

| Gene | Gender | Age | WhiteCells |
| --- | --- | --- | --- |
| SOX17 | Men | 0.0585 | -0.0124 |
|  | Women | 0.0242 | -0.0124 |

SOX17: Regression coefficients of continuous variables multiplied by their population standard deviations.

| 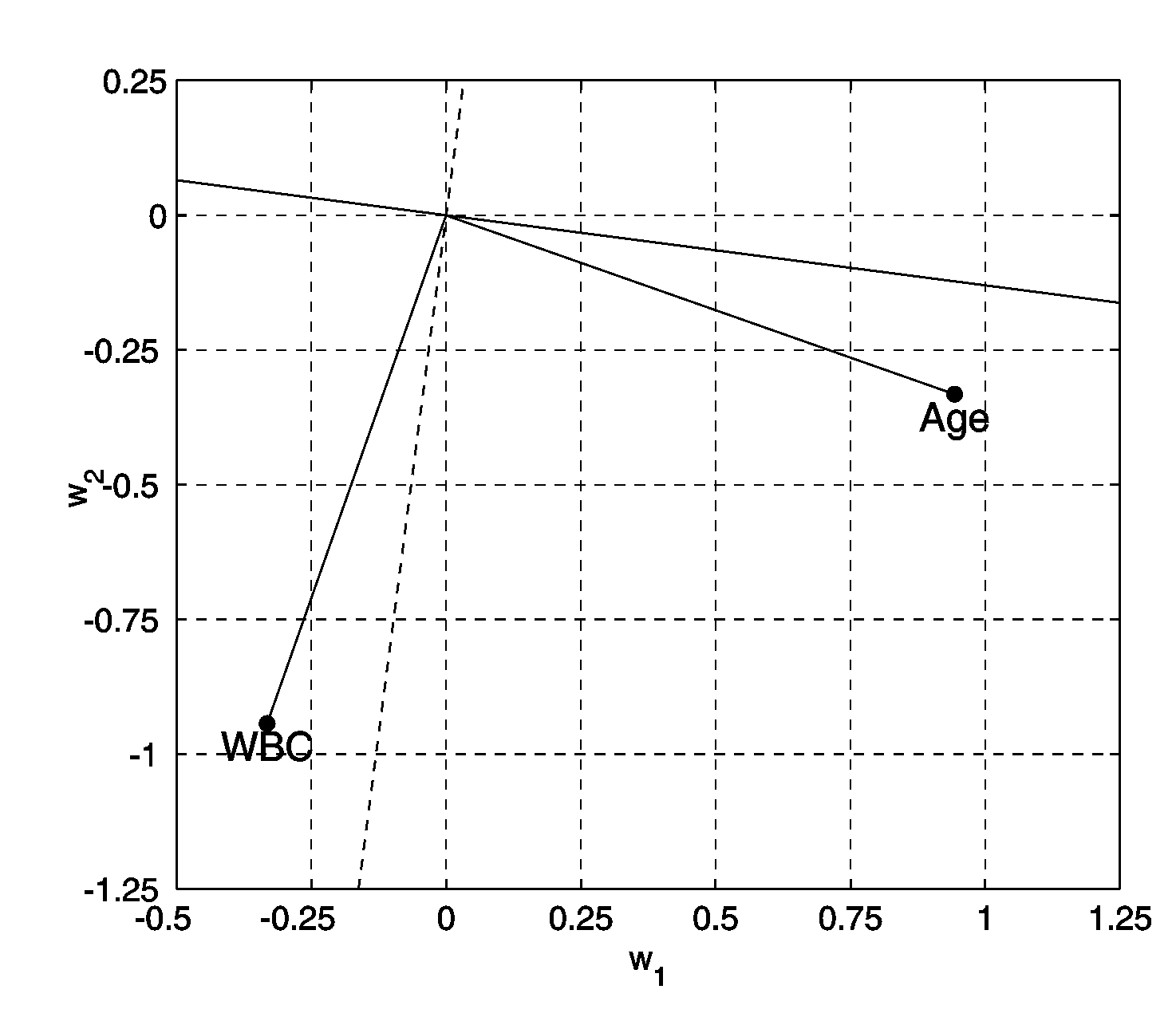 | 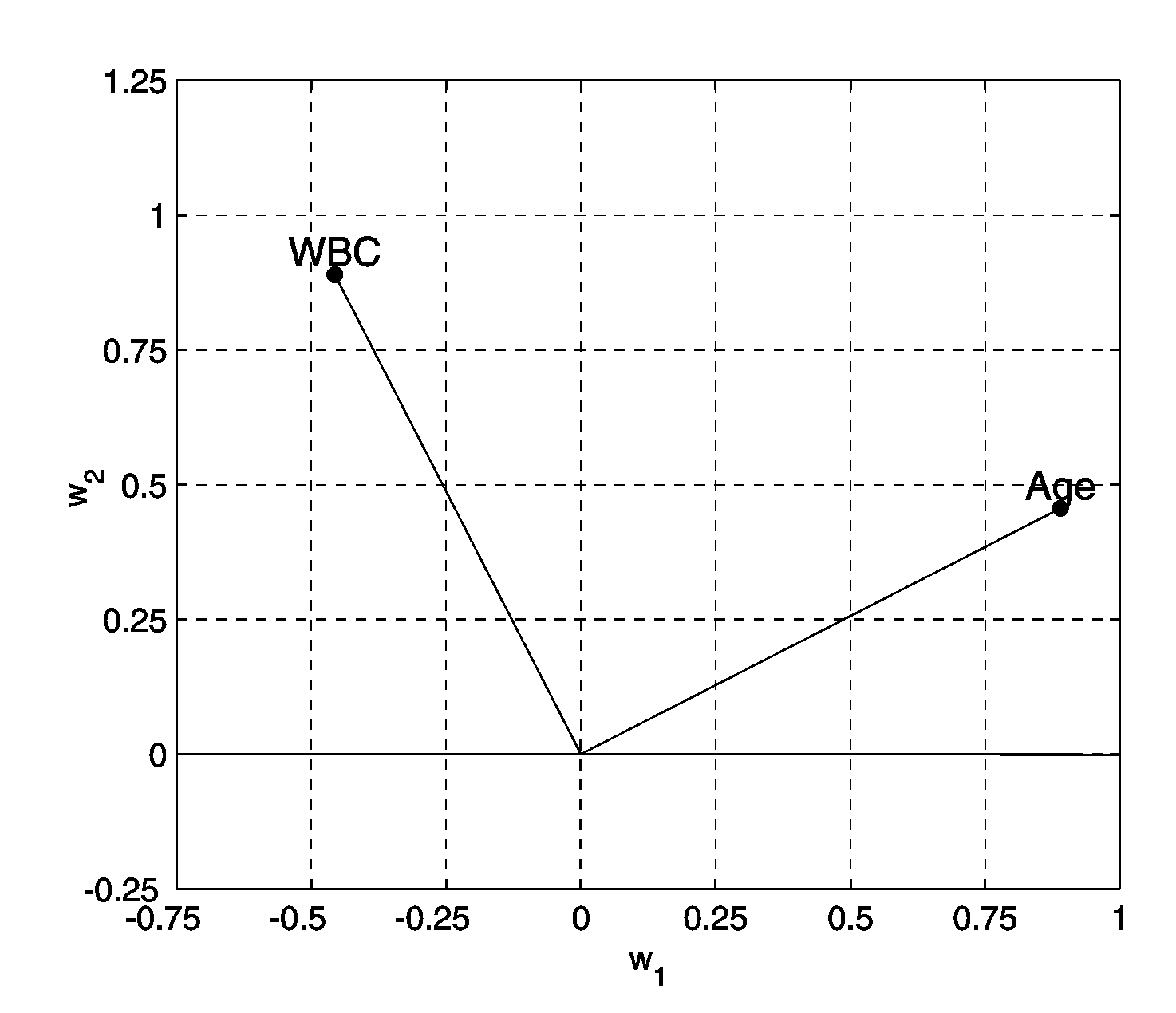 |
| --- | --- |
| Men: TinyLVR loading plot | Women: TinyLVR loading plot |

| **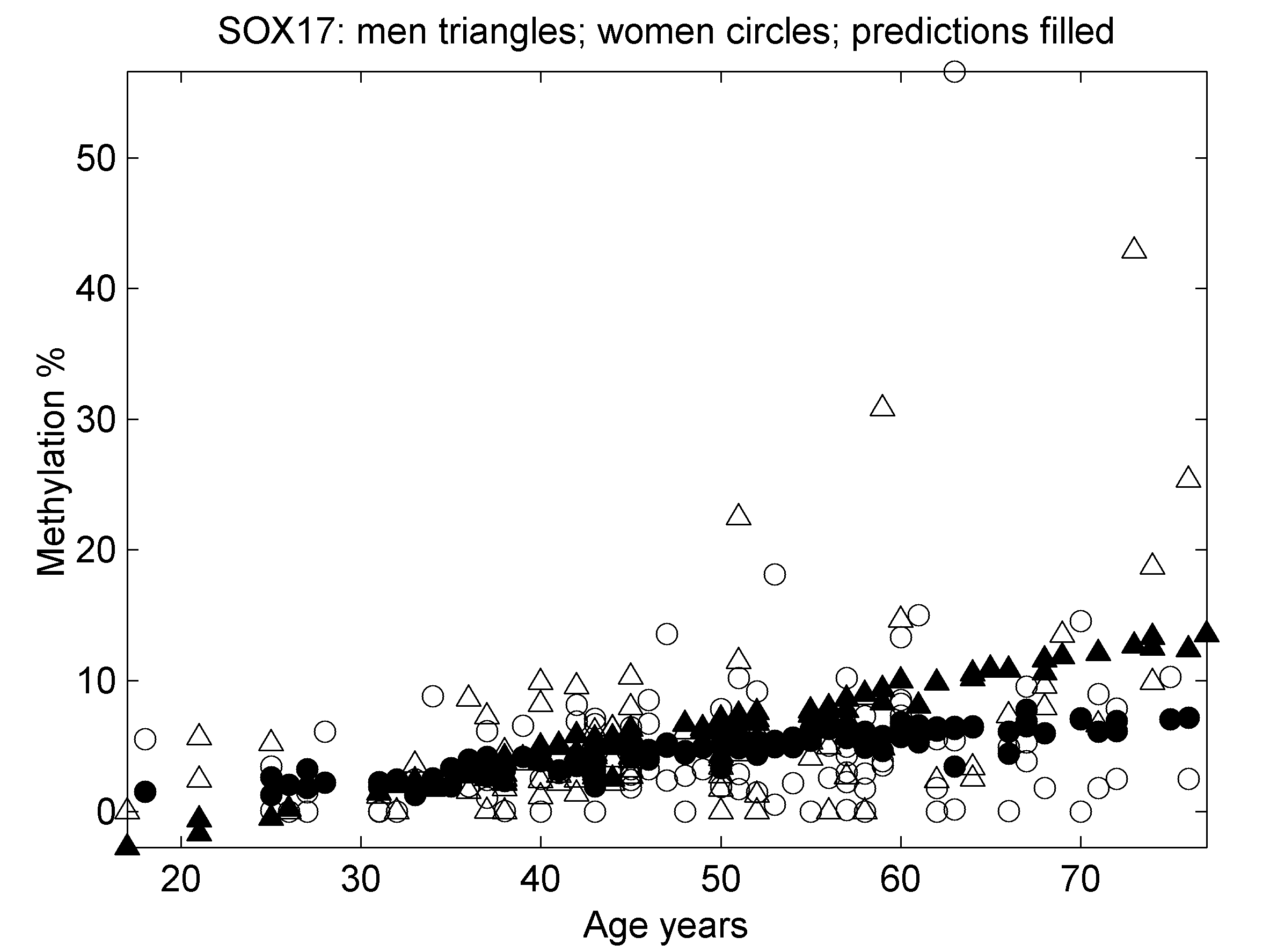** |
| --- |
| Variation in methylation with age: actual unscaled values and non-CV predictions |

**2.7 *WIF1***

| Source | Sum Sq. | d.f. | Mean Sq. | F | Prob>F |
| --- | --- | --- | --- | --- | --- |
| Age | 1.1864 | 1 | 1.1864 | 40.81 | 1.42E-09 |
| BMI | 0.1262 | 1 | 0.1262 | 4.34 | 0.0387 |
| SerumFol | 0.1231 | 1 | 0.1231 | 4.23 | 0.0411 |
| WhiteCells | 0.1489 | 1 | 0.1489 | 5.12 | 0.0248 |
| Moncyt | 0.2255 | 1 | 0.2255 | 7.76 | 0.0059 |
| Selenium | 0.2231 | 1 | 0.2231 | 7.67 | 0.0062 |
| Error | 5.1749 | 178 | 0.0291 |  |  |
| Total | 7.4173 | 184 |  |  |  |

WIF1 Type II ANCOVA table of GA selected model

| Gene | Gender | Age | BMI | SerumFol | WhiteCells | Moncyt | Selenium |
| --- | --- | --- | --- | --- | --- | --- | --- |
| WIF1 | Both | 0.0828 | 0.0265 | 0.0275 | -0.0338 | 0.0413 | -0.0365 |

WIF1: Regression coefficients of continuous variables multiplied by their population standard deviations.

| 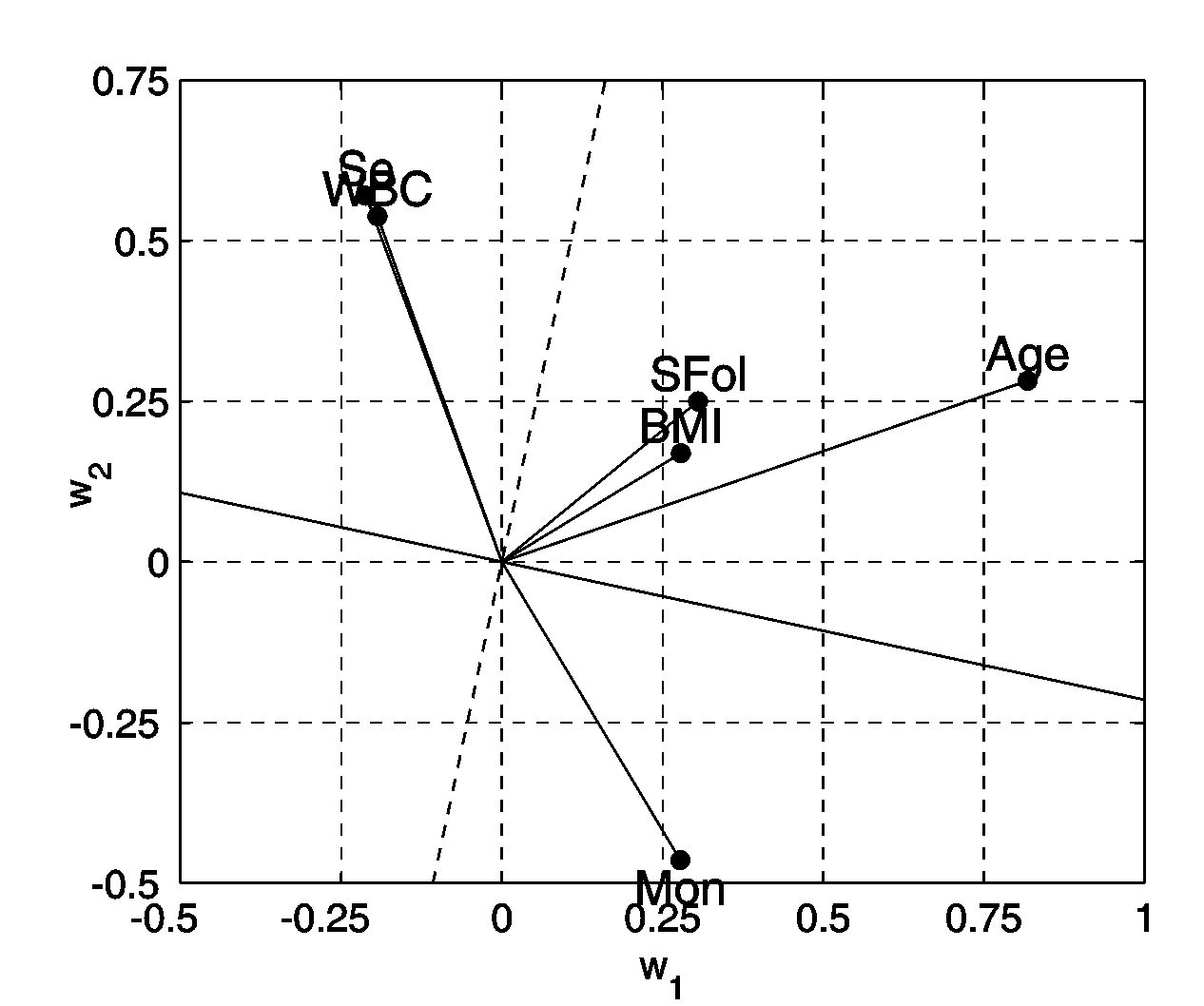 |
| --- |
| TinyLVR loading plot |

| **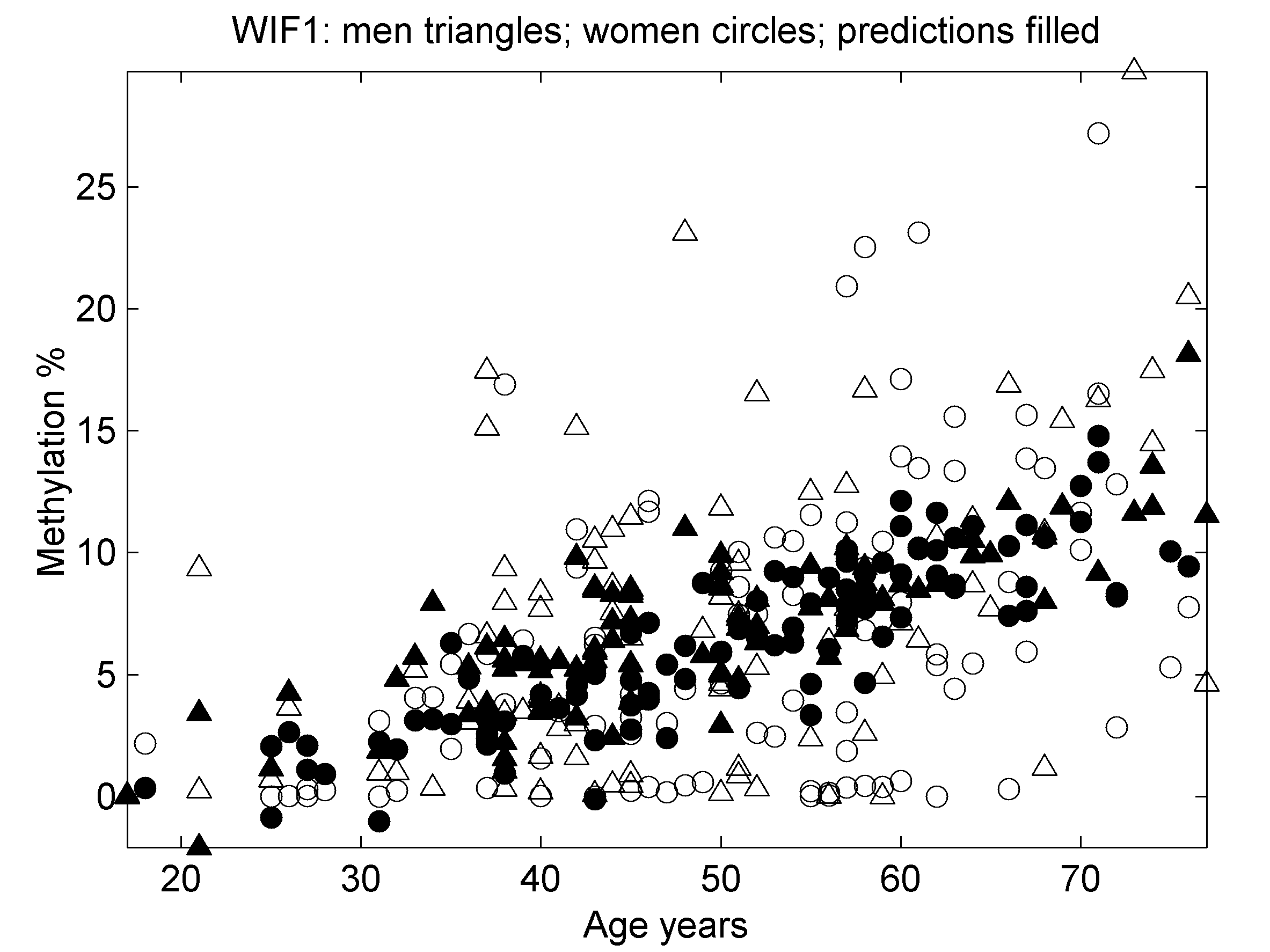** |
| --- |
| Variation in methylation with age: actual unscaled values and non-CV predictions |

**2.8 *ESR1***

| Source | Sum Sq. | d.f. | Mean Sq. | F | Prob>F |
| --- | --- | --- | --- | --- | --- |
| Age | 0.3286 | 1 | 0.3286 | 29.24 | 2.15E-07 |
| Sex | 0.00372 | 1 | 0.0037 | 0.33 | 0.5658 |
| Height | 0.0253 | 1 | 0.0253 | 2.25 | 0.1351 |
| Sex*Height | 0.0886 | 1 | 0.0886 | 7.88 | 0.0056 |
| Error | 1.8992 | 169 | 0.0112 |  |  |
| Total | 2.3203 | 173 |  |  |  |

ESR1 Type II ANCOVA table of GA selected model

| Gene | Gender | Age | Height |
| --- | --- | --- | --- |
| ESR1 | Men | 0.0469 | -0.0109 |
|  | Women | 0.0469 | 0.0598 |

ESR1: Regression coefficients of continuous variables multiplied by their population standard deviations.

| 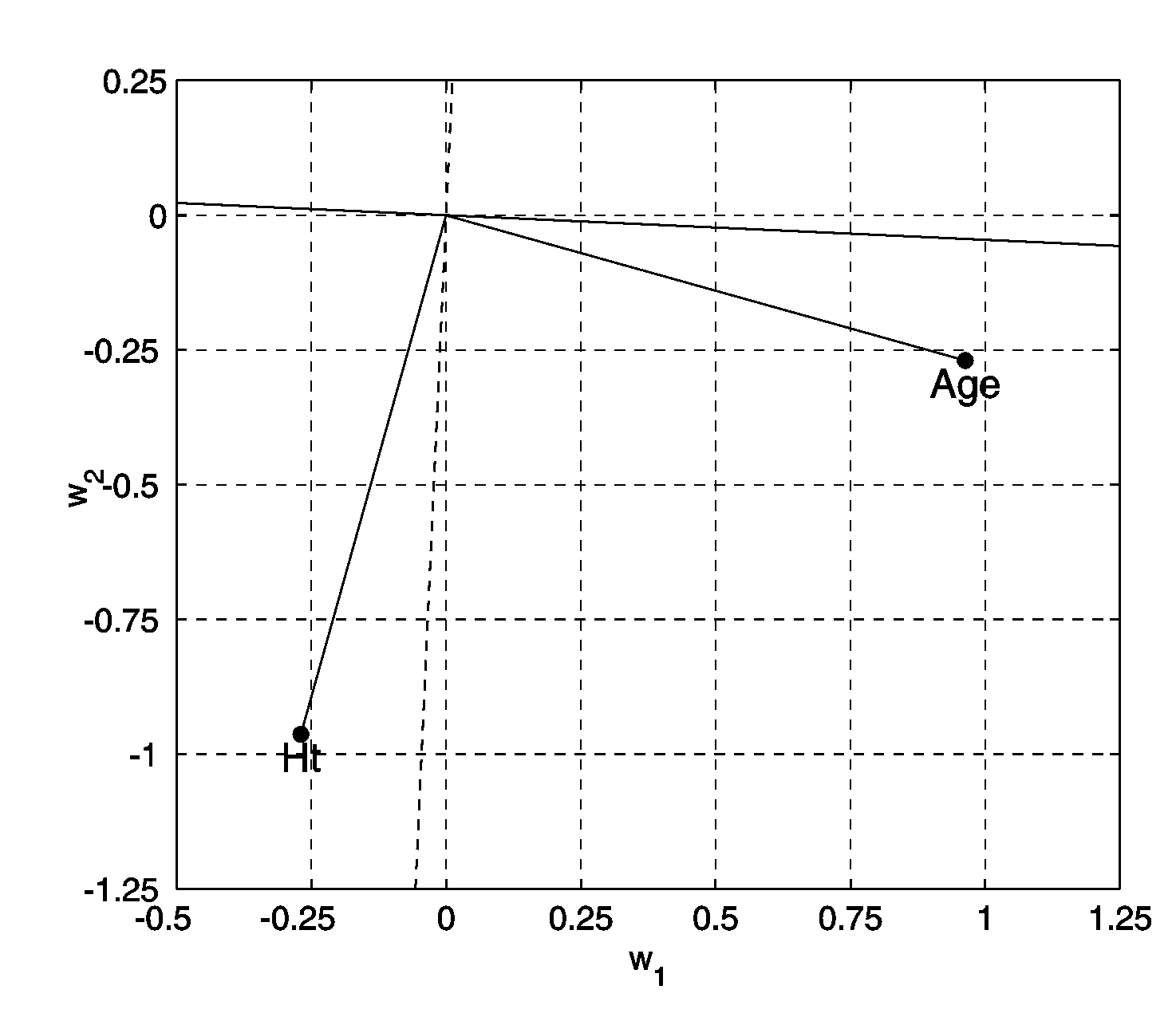 | 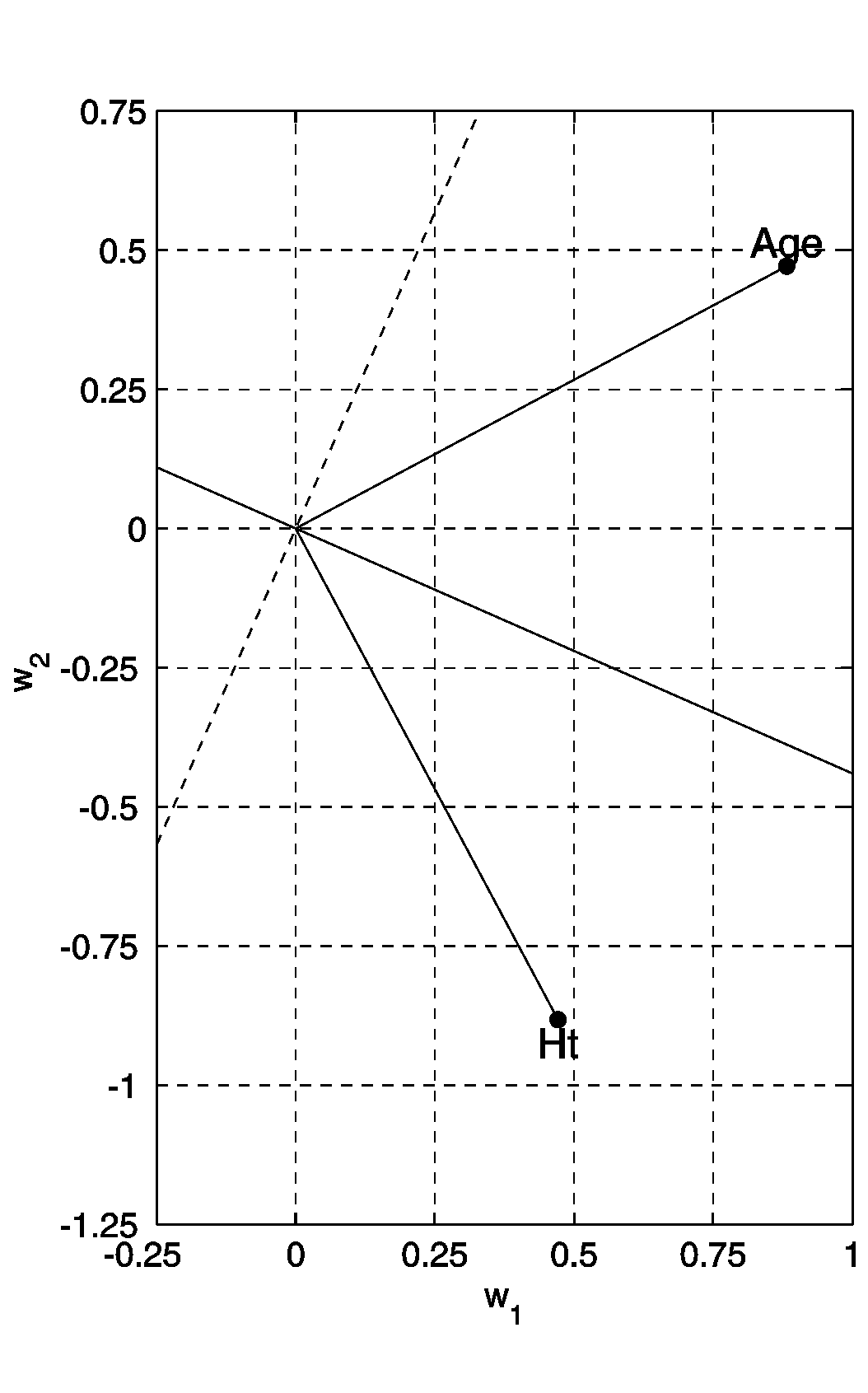 |
| --- | --- |
| Men: TinyLVR loading plot | Women: TinyLVR loading plot |

| **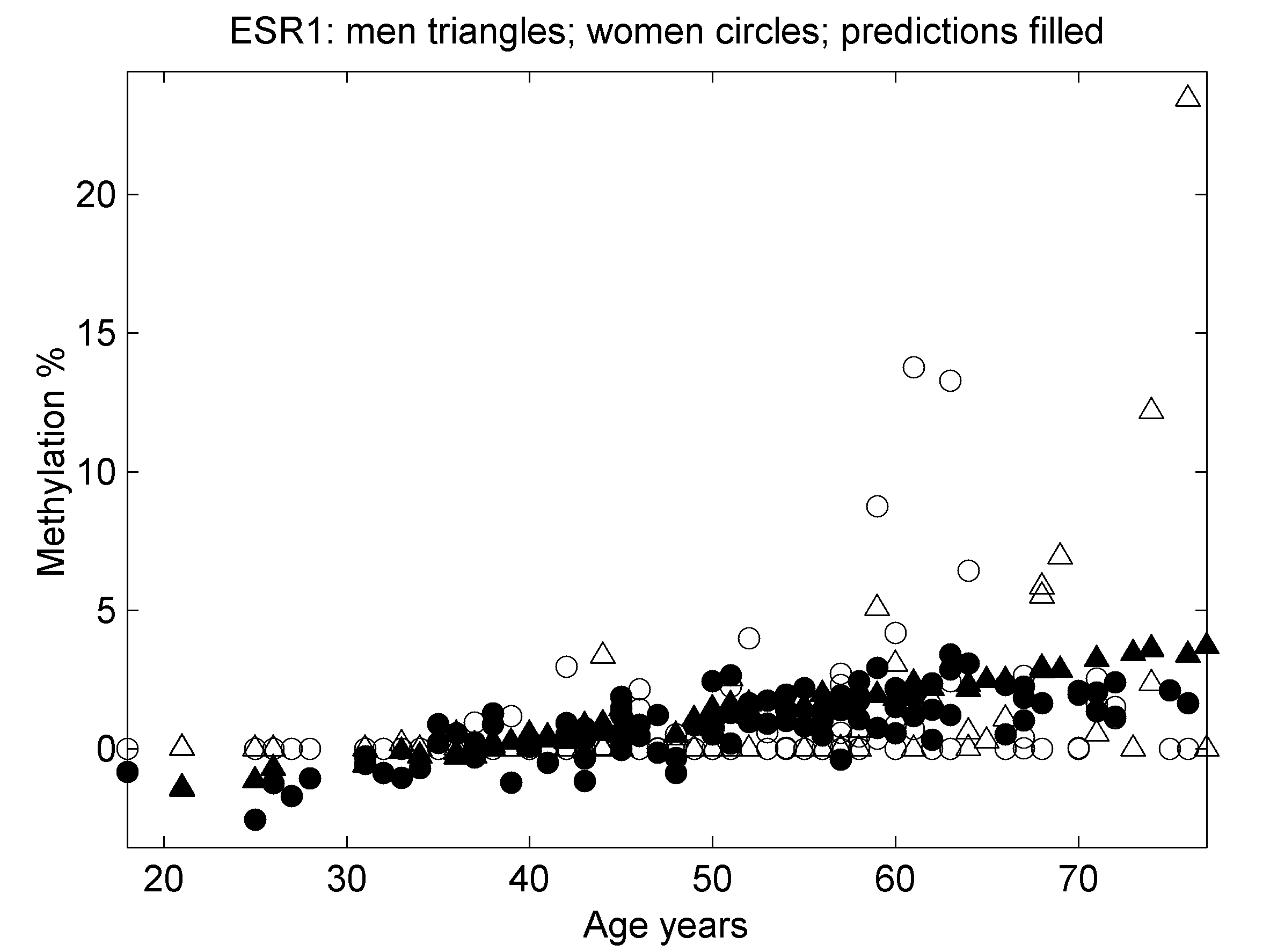** |
| --- |
| Variation in methylation with age: actual unscaled values and non-CV predictions |

**2.9 *MYOD***

| Source | Sum Sq. | d.f. | Mean Sq. | F | Prob>F |
| --- | --- | --- | --- | --- | --- |
| Age | 0.2183 | 1 | 0.2183 | 13.19 | 0.0004 |
| SerumFol | 0.1646 | 1 | 0.1646 | 9.95 | 0.0019 |
| VitD | 0.0641 | 1 | 0.0641 | 3.88 | 0.0506 |
| Error | 2.8131 | 170 | 0.0165 |  |  |
| Total | 3.3874 | 173 |  |  |  |

MYOD Type II ANCOVA table of GA selected model

| Gene | Gender | Age | SerumFol | VitD |
| --- | --- | --- | --- | --- |
| MYOD | Both | 0.0375 | 0.0313 | -0.0197 |

MYOD: Regression coefficients of continuous variables multiplied by their population standard deviations.

| 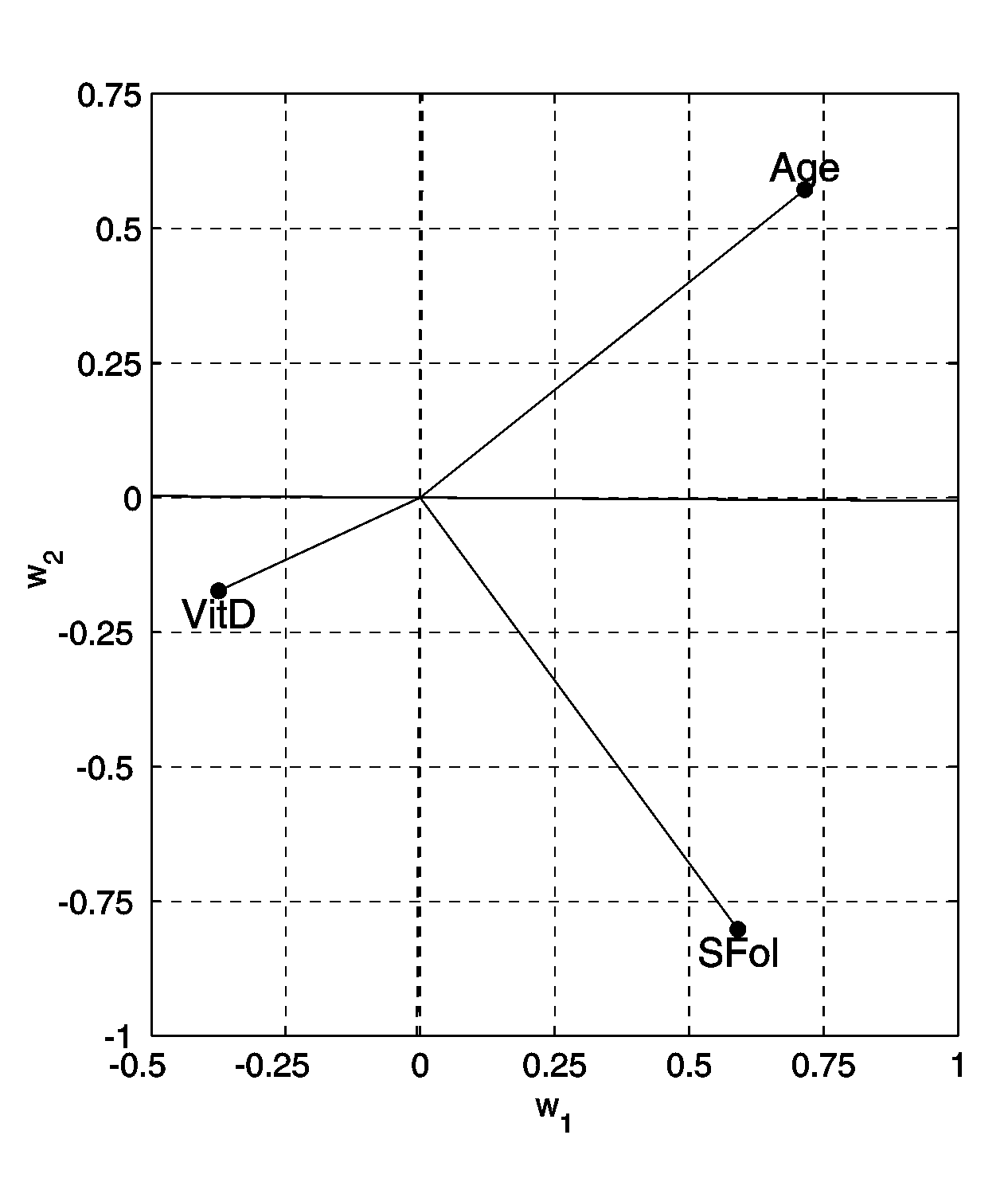 |
| --- |
| TinyLVR loading plot |

| **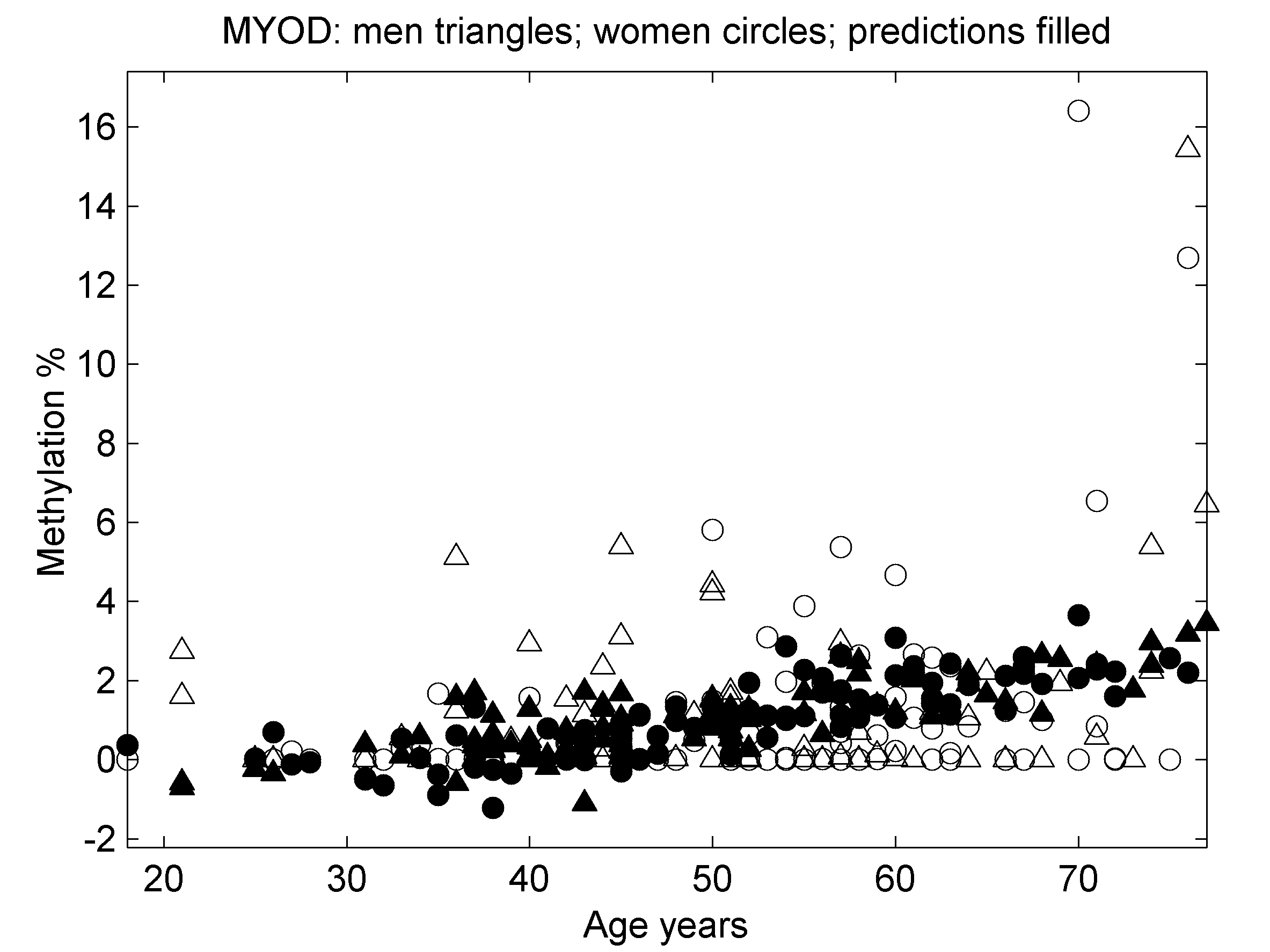** |
| --- |
| Variation in methylation with age: actual unscaled values and non-CV predictions |

**2.10 *N33***

| Source | Sum Sq. | d.f. | Mean Sq. | F | Prob>F |
| --- | --- | --- | --- | --- | --- |
| Age | 0.2653 | 1 | 0.2653 | 13.13 | 0.0004 |
| Sex | 0.0279 | 1 | 0.0279 | 1.38 | 0.2417 |
| Waist | 0.0207 | 1 | 0.0207 | 1.02 | 0.3134 |
| SerumFol | 0.0115 | 1 | 0.0115 | 0.57 | 0.4514 |
| Sex*Waist | 0.0826 | 1 | 0.0826 | 4.09 | 0.0447 |
| Sex*SerumFol | 0.1018 | 1 | 0.1018 | 5.04 | 0.0261 |
| Error | 3.3736 | 167 | 0.0202 |  |  |
| Total | 3.8815 | 173 |  |  |  |

N33 Type II ANCOVA table of GA selected model

| Gene | Gender | Age | Waist | SerumFol |
| --- | --- | --- | --- | --- |
| N33 | Men | 0.0411 | -0.0141 | -0.0367 |
|  | Women | 0.0411 | 0.0347 | 0.0123 |

N33: Regression coefficients of continuous variables multiplied by their population standard deviations.

| 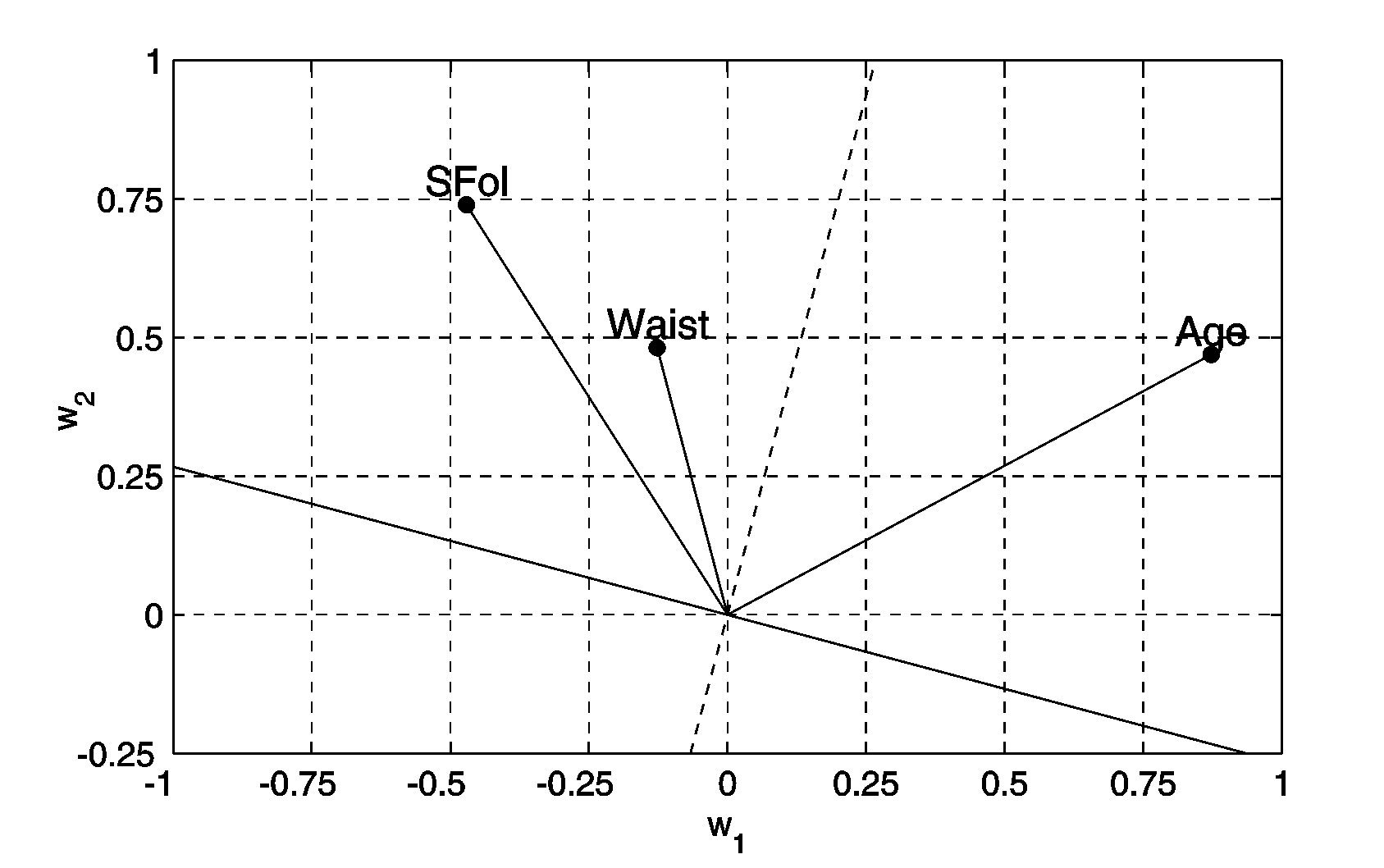 | 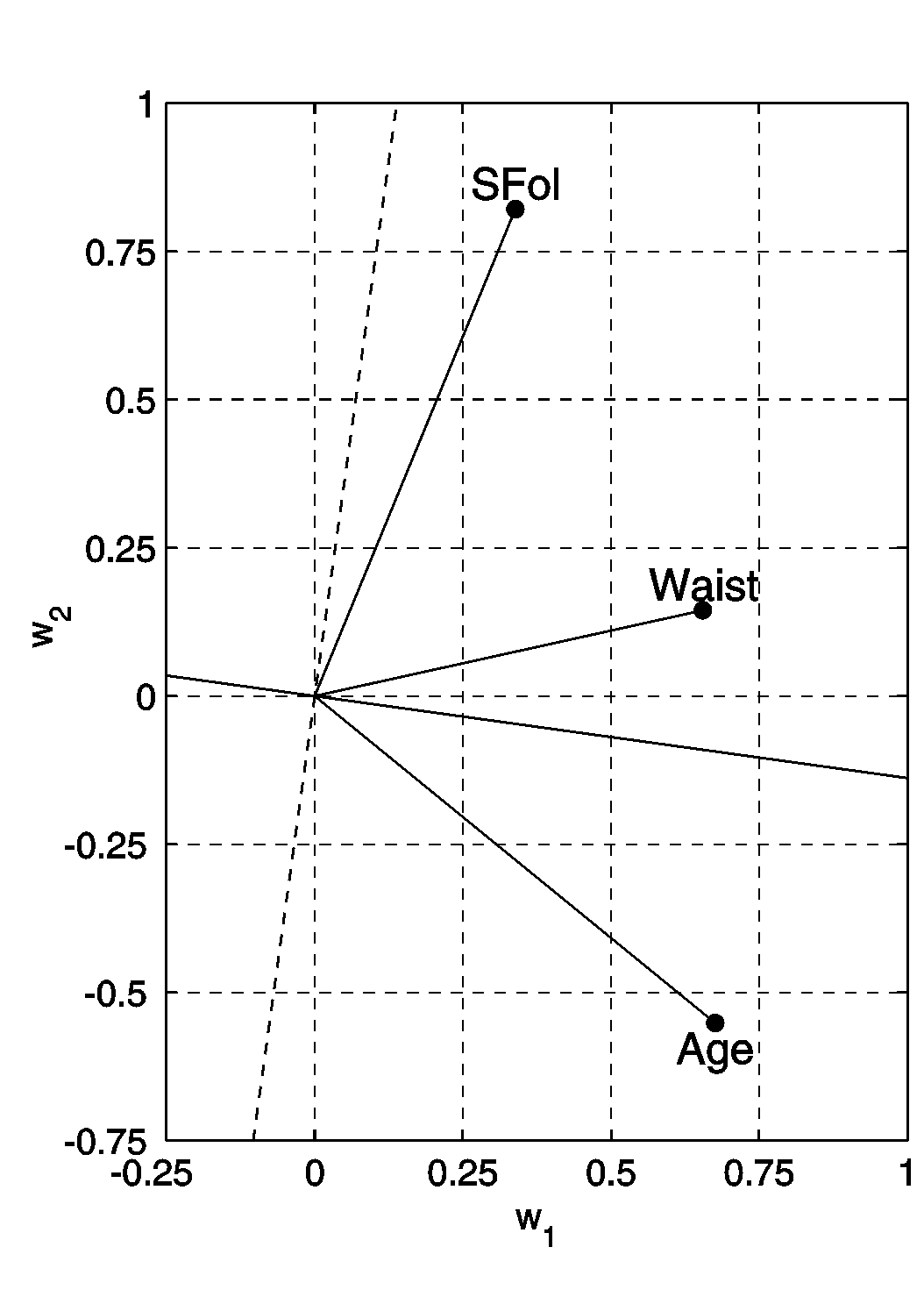 |
| --- | --- |
| Men: TinyLVR loading plot | Women: TinyLVR loading plot |

| **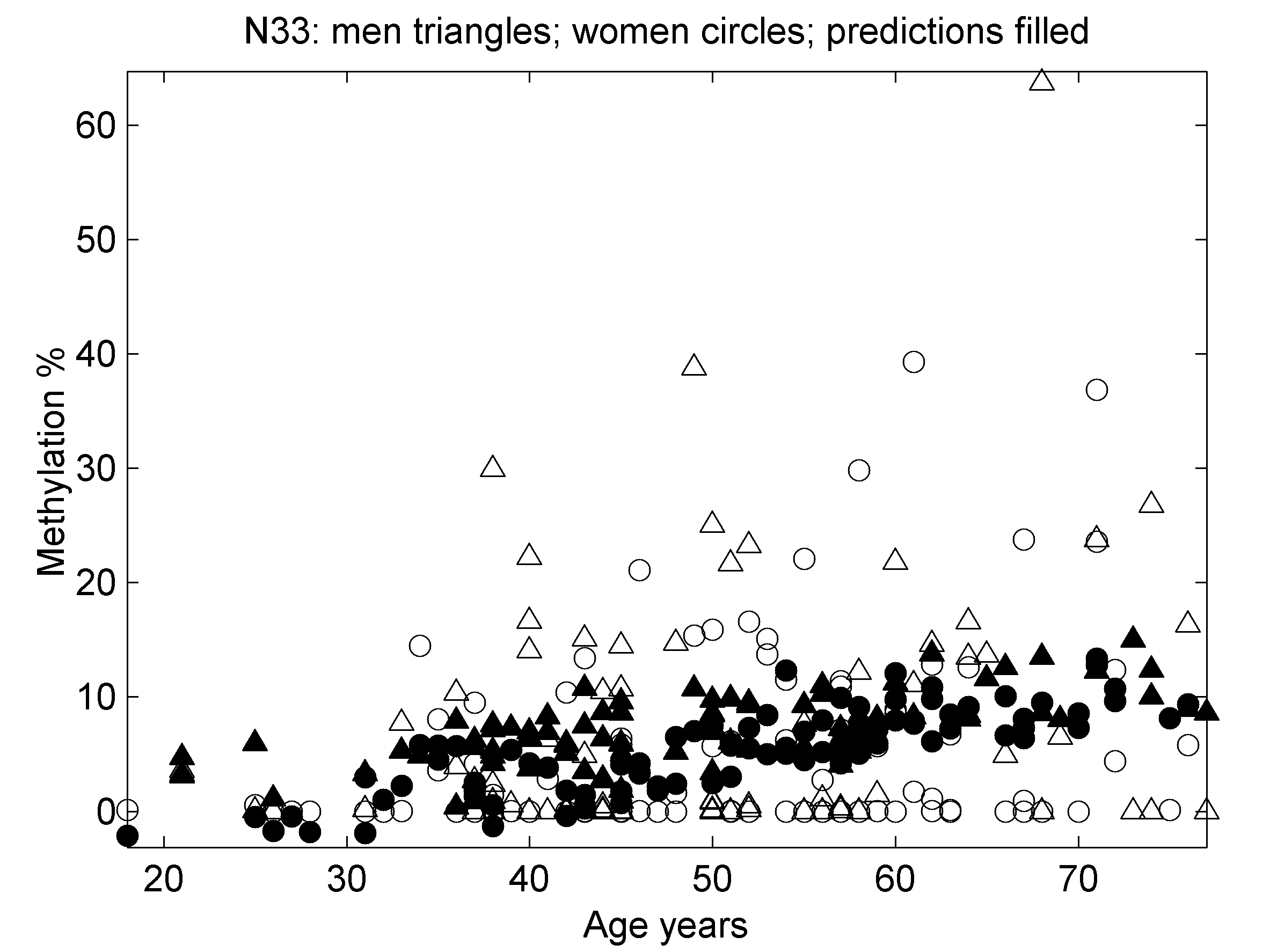** |
| --- |
| Variation in methylation with age: actual unscaled values and non-CV predictions |

**2.11 PCA1**

| Source | Sum Sq. | d.f. | Mean Sq. | F | Prob>F |
| --- | --- | --- | --- | --- | --- |
| Age | 1.0545 | 1 | 1.0545 | 70.87 | 1.66E-14 |
| Sex | 0.0629 | 1 | 0.0629 | 4.23 | 0.0413 |
| SerumFol | 0.0968 | 1 | 0.0968 | 6.50 | 0.0117 |
| VitD | 0.0225 | 1 | 0.0225 | 1.51 | 0.2205 |
| Selenium | 0.0310 | 1 | 0.0310 | 2.09 | 0.1505 |
| Age*Sex | 0.0771 | 1 | 0.0771 | 5.18 | 0.0241 |
| Error | 2.4848 | 167 | 0.0149 |  |  |
| Total | 4.0045 | 173 |  |  |  |

PCA1 Type II ANCOVA table of GA selected model

| Gene | Gender | Age | SerumFol | VitD | Selenium |
| --- | --- | --- | --- | --- | --- |
| PCA1 | Men | 0.1067 | 0.0246 | -0.0120 | -0.0142 |
|  | Women | 0.0626 | 0.0246 | -0.0120 | -0.0142 |

PCA1: Regression coefficients of continuous variables multiplied by their population standard deviations.

| 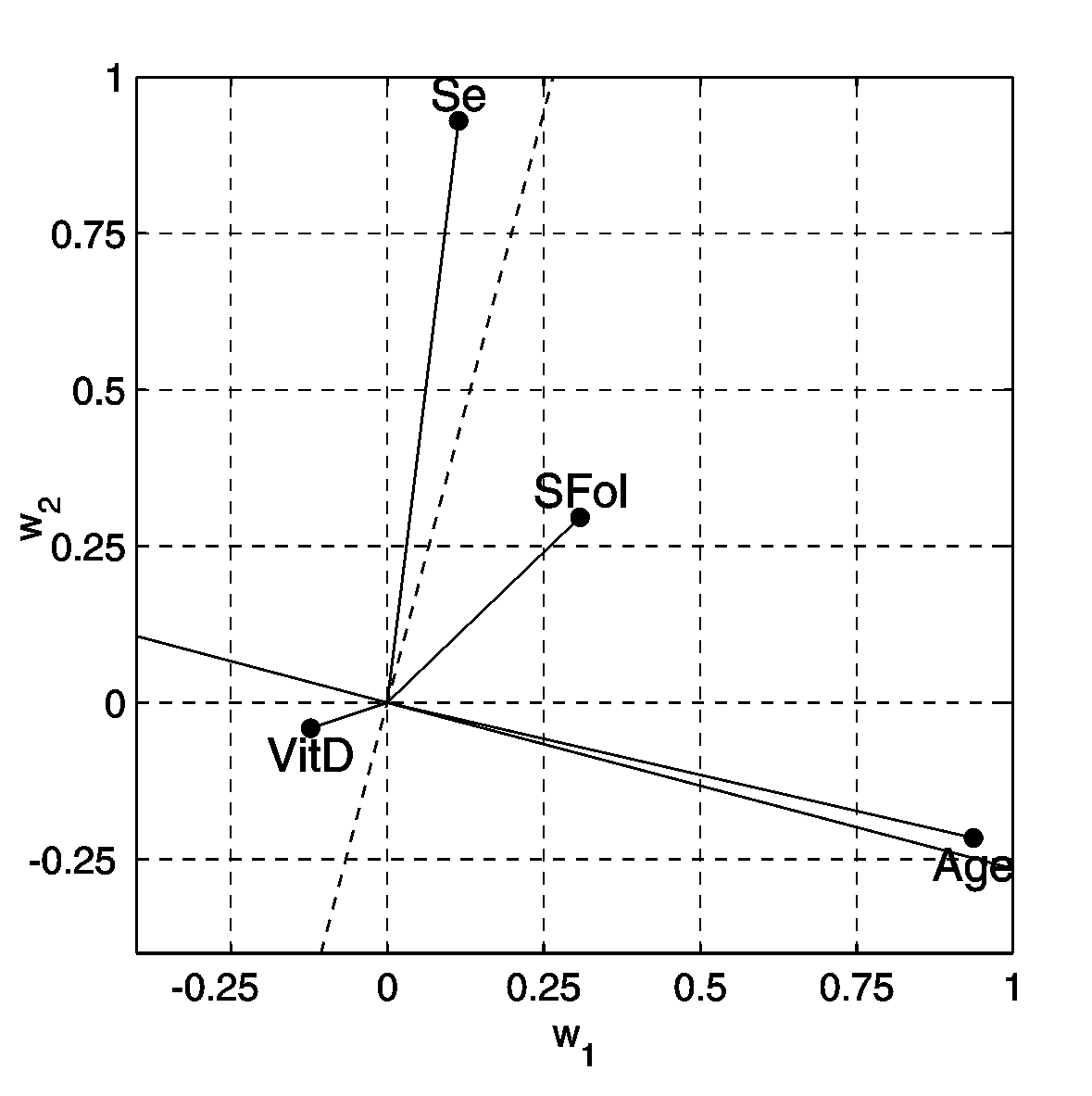 | 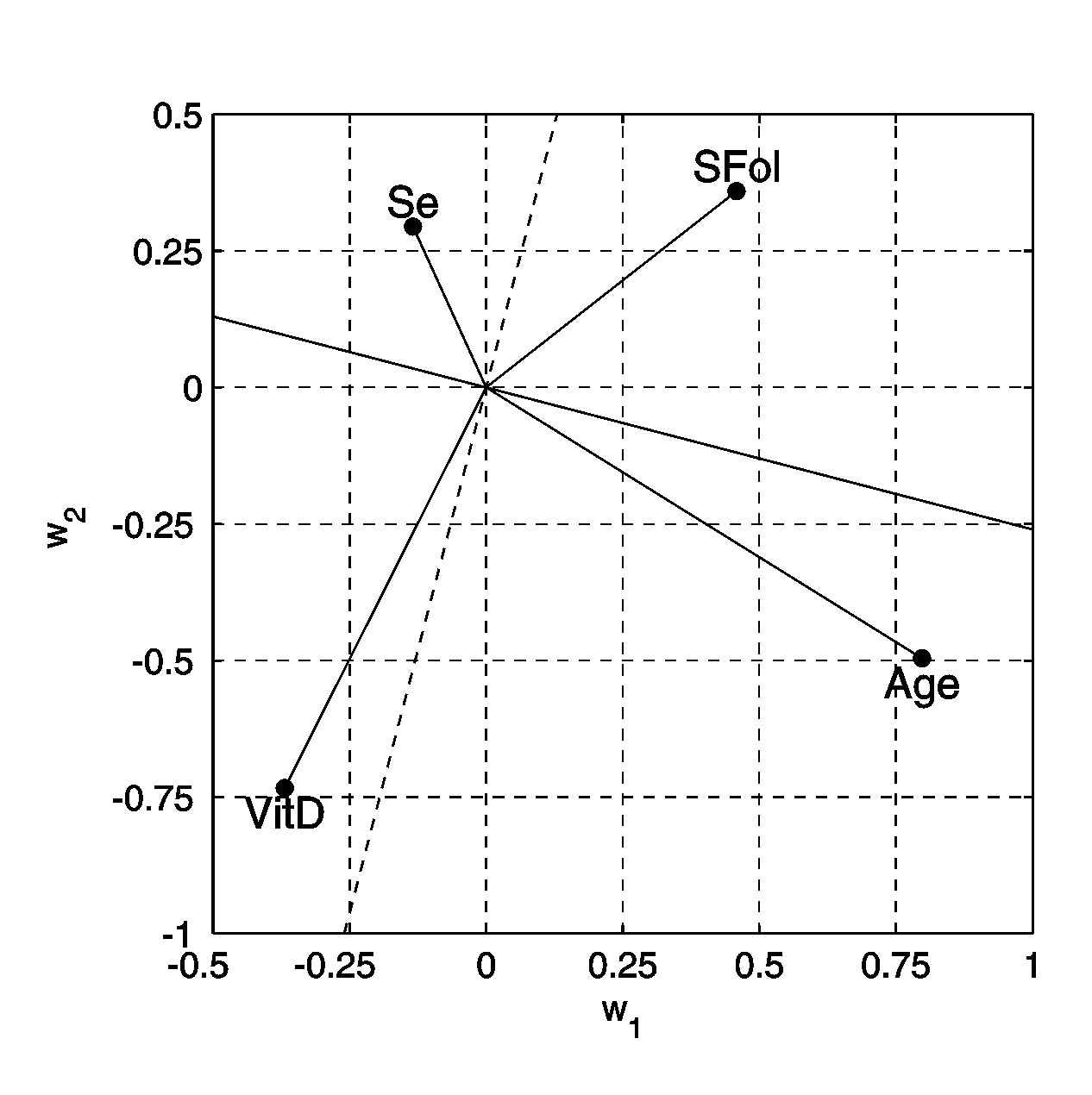 |
| --- | --- |
| Men: TinyLVR loading plot | Women: TinyLVR loading plot |

| **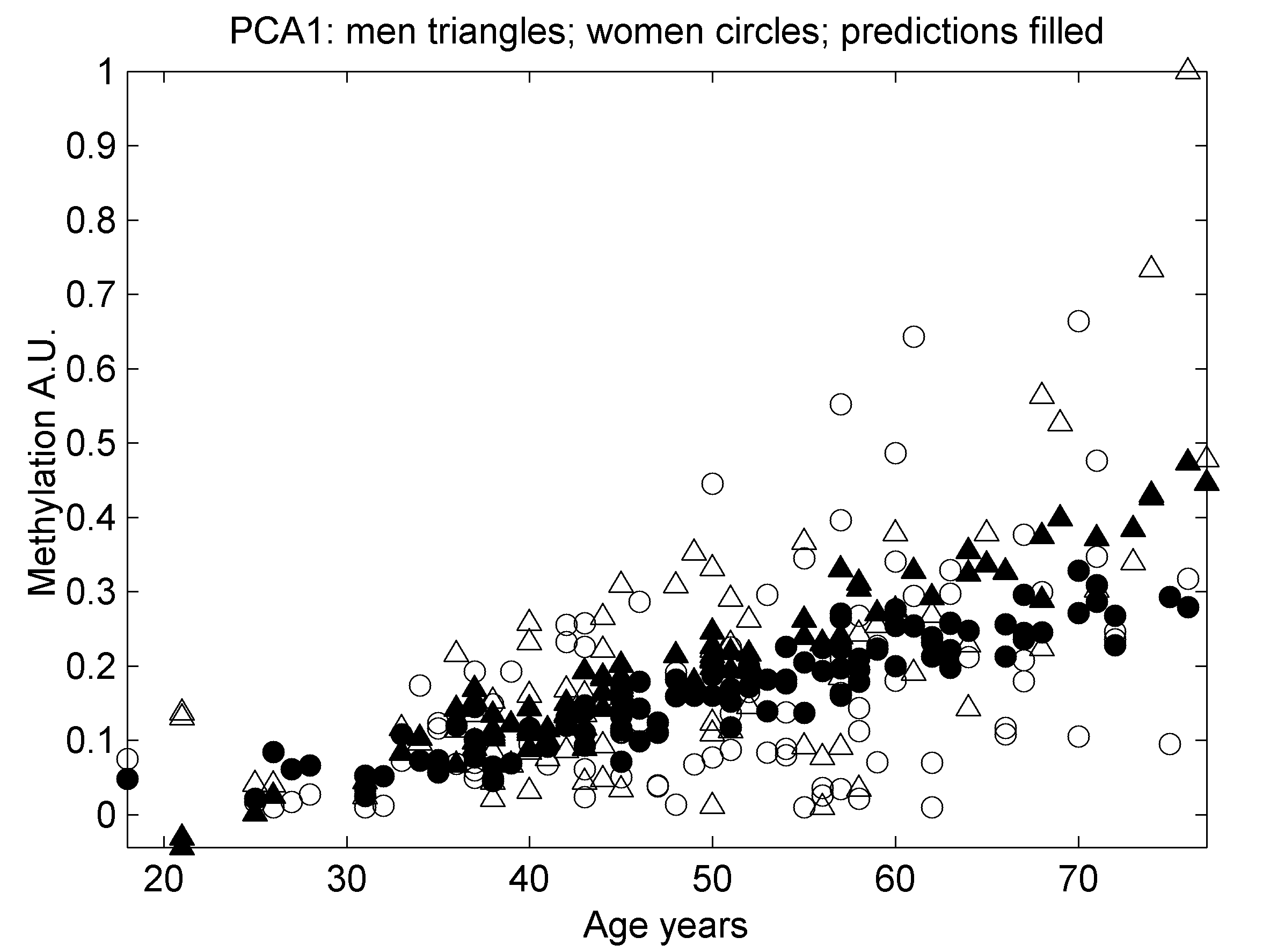** |
| --- |
| Variation in methylation with age: actual unscaled values and non-CV predictions |
