# Supplementary material for: Nutritional factors and gender influence age-related DNA methylation in the human rectal mucosa
Source: Aging Cell. 2012 Dec 6;12(1):148–55. doi: 10.1111/acel.12030 (PMC3572581; doi:10.1111/acel.12030)
Supplement: Supplementary file 3 [file acel0012-0148-sd3.doc]

| Correlation | Age | Sex | Height | Weight | BMI | Waist | Hip | WHR | RedCFol | SerumFol | HomoCyst | WhiteCells | Moncyt | VitD | Selenium | FI | B12 |
| --- | --- | --- | --- | --- | --- | --- | --- | --- | --- | --- | --- | --- | --- | --- | --- | --- | --- |
| Age |  | 0.092 | **-0.243**^3^ | -0.089 | 0.033 | 0.070 | 0.127 | -0.017 | **0.154**^1^ | **0.176**^1^ | 0.089 | -0.080 | 0.088 | **-0.210**^2^ | 0.098 | **0.213**^2^ | 0.042 |
| Sex | 0.092 |  | **-0.761^3^** | **-0.374**^3^ | 0.015 | **-0.380**^3^ | 0.044 | **-0.675**^3^ | -0.120 | 0.019 | -0.130 | 0.070 | **-0.188**^1^ | 0.104 | **0.162**^1^ | **0.647**^3^ | 0.082 |
| Height | **-0.243**^3^ | **-0.761^3^** |  | **0.529**^3^ | 0.026 | **0.405**^3^ | 0.118 | **0.521**^3^ | 0.038 | -0.086 | 0.109 | -0.140 | 0.063 | -0.056 | **-0.222**^2^ | **-0.419**^3^ | -0.127 |
| Weight | -0.089 | **-0.374**^3^ | **0.529**^3^ |  | **0.854**^3^ | **0.920**^3^ | **0.819**^3^ | **0.592**^3^ | 0.109 | -0.095 | 0.013 | -0.109 | 0.001 | -0.103 | **-0.208**^2^ | **0.389**^3^ | -0.123 |
| BMI | 0.033 | 0.015 | 0.026 | **0.854**^3^ |  | **0.837**^3^ | **0.908**^3^ | **0.373**^3^ | 0.093 | -0.064 | -0.050 | -0.041 | -0.048 | -0.077 | -0.116 | **0.716**^3^ | -0.061 |
| Waist | 0.070 | **-0.380**^3^ | **0.405**^3^ | **0.920**^3^ | **0.837**^3^ |  | **0.807**^3^ | **0.746**^3^ | 0.108 | -0.113 | 0.056 | -0.064 | 0.052 | **-0.194**^2^ | **-0.208**^2^ | **0.393**^3^ | -0.116 |
| Hip | 0.127 | 0.044 | 0.118 | **0.819**^3^ | **0.908**^3^ | **0.807**^3^ |  | **0.214**^2^ | 0.096 | -0.050 | -0.041 | -0.049 | -0.064 | -0.128 | -0.129 | **0.703**^3^ | -0.090 |
| WHR | -0.017 | **-0.675** | **0.521**^3^ | **0.592^3^** | **0.373**^3^ | **0.746**^3^ | **0.214**^2^ |  | 0.073 | -0.128 | 0.140 | -0.049 | 0.141 | **-0.181**^1^ | **-0.187**^1^ | -0.125 | -0.088 |
| RedCFol | **0.154**^1^ | -0.120 | 0.038 | 0.109 | 0.093 | 0.108 | 0.096 | 0.073 |  | **0.663**^3^ | **-0.235**^2^ | -0.111 | 0.021 | 0.000 | **0.244**^3^ | 0.010 | **0.308**^3^ |
| SerumFol | **0.176**^1^ | 0.019 | -0.086 | -0.095 | -0.064 | -0.113 | -0.050 | -0.128 | **0.663**^3^ |  | **-0.236**^2^ | **-0.165**^1^ | -0.050 | 0.065 | **0.270**^3^ | -0.014 | **0.303**^3^ |
| HomoCyst | 0.089 | -0.130 | 0.109 | 0.013 | -0.050 | 0.056 | -0.041 | 0.140 | **-0.235**^2^ | **-0.236**^2^ |  | -0.065 | -0.011 | -0.054 | **-0.256**^3^ | -0.128 | **-0.302**^3^ |
| WhiteCells | -0.080 | 0.070 | -0.140 | -0.109 | -0.041 | -0.064 | -0.049 | -0.049 | -0.111 | **-0.165**^1^ | -0.065 |  | **0.511**^3^ | -0.084 | -0.091 | -0.017 | -0.039 |
| Moncyt | 0.088 | **-0.188^1^** | 0.063 | 0.001 | -0.048 | 0.052 | -0.064 | 0.141 | 0.021 | -0.050 | -0.011 | **0.511**^3^ |  | -0.037 | -0.064 | **-0.176**^1^ | -0.095 |
| VitD | **-0.210**^2^ | 0.104 | -0.056 | -0.103 | -0.077 | **-0.194**^2^ | -0.128 | **-0.181^1^** | 0.000 | 0.065 | -0.054 | -0.084 | -0.037 |  | **0.159**^1^ | 0.003 | 0.020 |
| Selenium | 0.098 | **0.162^1^** | **-0.222**^2^ | **-0.208**^2^ | -0.116 | **-0.208**^2^ | -0.129 | **-0.187**^1^ | **0.244**^3^ | **0.270**^3^ | **-0.256**^3^ | -0.091 | -0.064 | **0.159**^1^ |  | 0.042 | **0.338**^3^ |
| FI | **0.213**^2^ | **0.647^3^** | **-0.419**^3^ | **0.389**^3^ | **0.716**^3^ | **0.393**^3^ | **0.703**^3^ | -0.125 | 0.010 | -0.014 | -0.128 | -0.017 | **-0.176**^1^ | 0.003 | 0.042 |  | 0.031 |
| B12 | 0.042 | 0.082 | -0.127 | -0.123 | -0.061 | -0.116 | -0.090 | -0.088 | **0.308**^3^ | **0.303**^3^ | **-0.302**^3^ | -0.039 | -0.095 | 0.020 | **0.338**^3^ | 0.031 |  |
| Mean | 49.557 | 0.092 | 1.694 | 81.909 | 28.454 | 93.082 | 105.289 | 0.882 | 382.670 | 7.245 | 11.850 | 7.999 | 0.543 | 78.825 | 1.112 | 31.940 | 408.743 |
| St. dev. | 13.080 | 0.998 | 0.104 | 20.359 | 6.033 | 15.775 | 12.264 | 0.090 | 168.365 | 3.732 | 7.847 | 2.271 | 0.185 | 42.925 | 0.252 | 7.820 | 172.343 |

Pearson correlations between cofactors. Correlations in bold have unadjusted p-values, p(r)<0.05. Superscripts denote the level of significant (unadjusted p-values): 1, p(r) < 0.05; 2, p(r) < 0.01; 3, p(r) < 0.001. Correlations with sex based on following coding scheme: men (-1), women (+1).
