# Supplementary material for: Nutritional factors and gender influence age-related DNA methylation in the human rectal mucosa
Source: Aging Cell. 2012 Dec 6;12(1):148–55. doi: 10.1111/acel.12030 (PMC3572581; doi:10.1111/acel.12030)
Supplement: Supplementary file 4 [file acel0012-0148-sd4.doc]

|  | LINE-1 | *HPP1* | *APC* | *SFRP1* | *SFRP2* | *SOX17* | *WIF1* | *ESR1* | *MYOD* | *N33* | PCA1 |
| --- | --- | --- | --- | --- | --- | --- | --- | --- | --- | --- | --- |
| LINE-1 |  | -0.070 | -0.029 | -0.064 | -0.090 | -0.040 | -0.138 | -0.119 | -0.027 | -0.056 | -0.128 |
| *HPP1* | -0.070 |  | 0.161^1^ | **0.484**^3^ | **0.409**^3^ | **0.435**^3^ | **0.357**^3^ | **0.468**^3^ | **0.419**^3^ | **0.316**^3^ | **0.742**^3^ |
| *APC* | -0.029 | 0.161^1^ |  | 0.238^2^ | 0.192^1^ | 0.063 | 0.133 | 0.200^2^ | 0.198^2^ | 0.210^2^ | 0.212^2^ |
| *SFRP1* | -0.064 | **0.484**^3^ | 0.238^2^ |  | **0.445**^3^ | **0.431**^3^ | **0.375**^3^ | **0.486**^3^ | **0.493**^3^ | **0.416**^3^ | **0.648**^3^ |
| *SFRP2* | -0.090 | **0.409**^3^ | 0.192^1^ | **0.445**^3^ |  | **0.303**^3^ | **0.251**^3^ | **0.321**^3^ | **0.471**^3^ | **0.424**^3^ | **0.513**^3^ |
| *SOX17* | -0.040 | **0.435**^3^ | 0.063 | **0.431**^3^ | **0.303**^3^ |  | **0.358**^3^ | **0.378**^3^ | **0.286**^3^ | 0.199^2^ | **0.561**^3^ |
| *WIF1* | -0.138 | **0.357**^3^ | 0.133 | **0.375**^3^ | **0.251**^3^ | **0.358**^3^ |  | **0.409**^3^ | **0.328**^3^ | 0.237^2^ | **0.721**^3^ |
| *ESR1* | -0.119 | **0.468**^3^ | 0.200^2^ | **0.486**^3^ | **0.321**^3^ | **0.378**^3^ | **0.409**^3^ |  | **0.281**^3^ | **0.310**^3^ | **0.623**^3^ |
| *MYOD* | -0.027 | **0.419**^3^ | 0.198^2^ | **0.493**^3^ | **0.471**^3^ | **0.286**^3^ | **0.328**^3^ | **0.281**^3^ |  | **0.494**^3^ | **0.524**^3^ |
| *N33* | -0.056 | **0.316**^3^ | 0.210^2^ | **0.416**^3^ | **0.424**^3^ | 0.199^2^ | 0.237^2^ | **0.310**^3^ | **0.494**^3^ |  | **0.445**^3^ |
| PCA1 | -0.128 | **0.742**^3^ | 0.212^2^ | **0.648**^3^ | **0.513**^3^ | **0.561**^3^ | **0.721**^3^ | **0.623**^3^ | **0.524**^3^ | **0.445**^3^ |  |

Spearman correlation coefficients between genes, LINE-1 and PCA1 based on 174 subjects. Figures in bold pass Dunn-Sidak test correction for 55 pairwise comparisons (i.e. have p < 9.32E-04). Superscripts refer to uncorrected significance level: 1, p<0.05; 2, p<0.01; 3, p<0.001.
