# Supplementary material for: Nutritional factors and gender influence age-related DNA methylation in the human rectal mucosa
Source: Aging Cell. 2012 Dec 6;12(1):148–55. doi: 10.1111/acel.12030 (PMC3572581; doi:10.1111/acel.12030)
Supplement: Supplementary file 6 [file acel0012-0148-sd6.doc]

**Supplementary Table S4.** Primer sequences and annealing temperatures for the initial amplification of each CGI from bisulphite-modified DNA and for each QMSP T and M reaction. For LINE-1, the qPCR unmethylated (U) and methylated (M) primer sequences are shown.

| Gene | PCR | Forward (5’ – 3’) | Reverse (5’ – 3’) | Position relative to tsp (bp) | Annealing T (^o^C) |
| --- | --- | --- | --- | --- | --- |
| *APC*  *ESR1*  *HPP1*  *SFRP2*  *MYOD1*  *N33*  *AXIN2*  *DKK1*  *WIF1*  *SFRP1*  *SOX17*  LINE-1 | Initial  T  M  Initial  T  M  Initial  T  M  Initial  T  M  Initial  T  M  Initial  T  M  Initial  T  M  Initial  T  M  Initial  T  M  Initial  T  M  Initial  T  M  U  M | GTTAGGGTTAGGTAGGTTGT  GGGTGTTATTGGAGATAGAAT  TATTGCGGAGTGCGGGTC  GGGATGGTTTTATTGTATTAGATTTAAGGG  GTAGTTTAAGATTTTTTTGGAG  GCGAGGTGTATTTGGATAGTAGTAAGTTCGTC  AGAGTTTTTTTTTTATGGTAGTAGTT  AGAGTTTTTTTTTTATGGTAGTAGTT  GTTTTTCGCGTTTTCGGCGT  GGTTAAGAAAATTTTGGTTGTG  GATGTTGTAGGGTTTGGTT  CGCGTTGTTTTTTCGGTGT  TAGGGGATAGAGGAGTATTG  GGGTTGGTTGGTTAGTTT  CGTTTTTCGCGGATATAG  TGGGTTTAGTAGTAGGATGG  GTGGAGGAGATATTGTTTGT  TACGTCGCGTTTTCGAAG  TTAGGATTTTTTGAATTTTTAAGGAGT  TTAGGATTTTTTGAATTTTTAAGGAGT  GTAGGGTCGAGTGGGGAAATATATC  TTTAGAAGGATTTAAGAGGGAGAAAG  GTTTTTTGGGAGGGAGATAA  GGGTTTTTACGAAATCGTGTC  GAGTGATGTTTTAGGGGTTT  GTATTGTGAATGTAGTTT  GGCGTTTTATTGGGCGTATC  GTTTTTTAAGGGGTGTTGAGT  GGGAGTTGATTGGTTG  CGGTCGTAGGAGTTTCGC  GGGGATATGAAGGTGAAG  GTTTTTATGGTGTGGGTTAAG  CGTATTCGGCGGTCGATGAAC  TGTGTGTGAGTTGAAGTAGGGT  CGCGAGTCGAAGTAGGGC | CCATAATAACTCCAACACCTA  CCATAATAACTCCAACACCTA  TCGACGAACTCCCGACGA  CTATTAAATAAAAAAAAACCCCCCAAAC  AACTTACTACTATCCAAATACACCTC  GTAAAAAAAACCGATCTAACCGTAAACCTACG  ACTCCCACAACACCATAACTA  AACATCCAAAAACTAAACTCAA  ATCATCCCGCGAACGACGA  ACCTACTCCAACACCTCCTTC  AACCCAACAAATTAA ACA ACC  CGCTCTCTTCGCTAAATACGACT  TTCCTCACCCCTAACTTCT  TTCCTCACCCCTAACTTCT  ACGACCGACAACCCTA  AAAAATCCATTCTACCTCCT  TTTTTCTTCTATCCTCCCC  TACGCGCCCAACTCCTA  AATATAAAACACAACCTTCCAAAAAC  CTAAACTATATACAAATAAAACCAATTTC  GATACCATCAACTCCAAAAAAACG  AAATAAAAAATATCAAAAACCCCC  AAATAAAAAATATCAAAAACCCCC  AAAAAACCGAAATACTCCGAA  AACCTAAATACCAAAAAACCTAC  TAAAACTAACAAAACTAAC  CTAACGAAACCAACAATCAACG  CAAACTTCCAAAAACCTCC  CAAACTTCCAAAAACCTCC  GACTCCCGAAAATACGACG  CACCCAACATCTTACTCAACT  CACCCAACATCTTACTCAACT  CTACGCCAACCGCTTACGCTCG  ACCCAATTTTCCAAATACAACCATCA  ACCCGATTTTCCAATACGACCG | -207 to +60  -9 to +60  -163 to -66  +392 to +666  +448 to +507  +480 to +601  +119 to +409  +119 to +215  +142 to +315  -28 to +467  +240 to +436  +68 to +158  -361 to +29  -163 to +29  -308 to -218  -158 to +362  +185 to +343  -57 to +172  -603 to -305  -603 to -434  -487 to -398  -299 to +15  -190 to +15  -196 to -42  -436 to -21  -349 to -153  -369 to -167  -182 to +231  -66 to +231  -29 to +70  +321 to +513  +423 to +513  +402 to +467 | 59.5  59  65  58  58  66  56  58  67  58  62  63  59  58  60  55  58  60  54  60  63  56  60  64  54  42  64  59  56  63  61  60  69  60  60 |
